# Mapping the genetic landscape of iron metabolism uncovers the SETD2 methyltransferase as a modulator of iron flux

**DOI:** 10.1126/sciadv.adw9095

**Published:** 2025-09-17

**Authors:** Anthony W. Martinelli, Chun-Pei Wu, Tristan Vornbäumen, Hudson W. Coates, Louise H. Jordon, Niek Wit, Jia J. Sia, Anneliese O. Speak, James A. Nathan

**Affiliations:** Cambridge Institute of Therapeutic Immunology & Infectious Disease (CITIID), Jeffrey Cheah Biomedical Centre, Cambridge Biomedical Campus, Department of Medicine, University of Cambridge, Cambridge CB2 0AW, UK.

## Abstract

Cellular iron levels must be tightly regulated to ensure sufficient iron for essential enzymatic functions while avoiding the harmful generation of toxic species. Here, to better understand how iron levels are controlled, we carry out genome-wide mutagenesis screens in human cells. Alongside mapping known components of iron sensing, we determine the relative contributions of iron uptake, iron recycling, ferritin breakdown, and mitochondrial flux in controlling the labile iron pool. We also identify SETD2, a histone methyltransferase, as a chromatin modifying enzyme that controls intracellular iron availability through ferritin breakdown. Functionally, we show that SETD2 inhibition or cancer-associated *SETD2* mutations render cells iron deficient, thereby driving resistance to ferroptosis and potentially explaining how some tumors evade antitumoral immunity.

## Teaser

Genetic screens using endogenous reporters map key modulators of cellular iron, including the transcriptional regulator SETD2.

## INTRODUCTION

Strict regulation of cellular iron levels is essential. Sufficient iron must be present within cells to ensure that it can be incorporated into life-critical proteins and facilitate redox reactions while avoiding the generation of harmful reactive oxygen species. Cells must therefore be able to sense and respond rapidly to changes in iron abundance.

Iron sensing is thought to be principally mediated at a posttranscriptional level by the iron regulatory proteins 1 and 2 (IRP1 and IRP2) (*1*–*5*). Under conditions of iron depletion, IRPs bind to iron-responsive elements (IREs) within the 5′ or 3′ regions of gene transcripts involved in iron metabolism. Binding within the 5′ region inhibits translation, whereas 3′ IREs promote mRNA stabilization and increased translation (*6*–*11*). The coordination of this IRP response maintains iron homeostasis by regulating pathways critical for iron uptake, recycling, and storage (*12*).

Most cellular iron is taken up in a transferrin (Tf)–bound ferric (Fe^3+^) form via the Tf receptor (TfR) (*13*). Iron is then converted to a ferrous (Fe^2+^) form in early endosomes before incorporation into proteins or delivery to the mitochondria (*14*–*16*). Excess iron is either exported from the cell, via ferroportin, or stored bound to ferritin—a protective cage that mitigates against the risks of this reactive metal (*17*–*20*). Reactive iron can be released from ferritin via a specialized form of autophagy, termed ferritinophagy, which requires a specific cargo receptor, NCOA4 (*21*).

Aside from the essential role of iron in sustaining cellular redox reactions, work over the past decade has identified that iron is necessary for a specialized form of cell death, ferroptosis, which is characterized by a lipid peroxidation cascade (*22*–*25*). Inducing ferroptosis provides a promising therapeutic approach to treat cancers, and it is also noteworthy that ferroptosis can be induced by CD8+ T cells as an antitumor mechanism (*26*–*29*). Furthermore, induction of ferroptosis in cancer cells up-regulates the expression of UL16 binding proteins (ULBPs), which are activating ligands for natural killer (NK) cells via the NKG2D receptor (*30*). Thus, there is potential for a synergistic effect both directly and indirectly via activation of innate and adaptive cytotoxic immune cells.

Although the relationship between iron uptake and ferritin breakdown is established, many aspects of cellular iron metabolism remain unknown, including the exact nature of free iron within the cell [the labile iron pool (LIP)] and how iron is trafficked and incorporated into proteins (*12*). The role of the nucleus in coordinating these processes is of particular interest, with recent works having identified a TfR-independent mechanism of cellular iron uptake via the CD44 transmembrane glycoprotein. CD44 is regulated at a transcriptional level by nuclear iron and a nuclear iron sensing pathway that acts via histone demethylation to regulate mTORC1 and anabolic metabolism (*31*–*33*). Demonstrating the existence of other epigenetic modulators of iron homeostasis could challenge the traditional model of cellular iron regulation as a purely posttranscriptional process. Lastly, whether the dysregulation of iron homeostasis that occurs in cancers protects against ferroptosis remains unclear, although recent works describe a role for modulating lysosomal iron as a potential therapeutic avenue in this context (*32*, *34*–*37*).

Here, we establish sensitive and dynamic reporters for measuring cellular iron flux and use these tools to identify the key genes and pathways regulating iron metabolism. Alongside mapping the landscape of cellular iron homeostasis, we uncover the histone methyltransferase SETD2 as a regular of cellular iron flux. We also show that *SETD2* mutations, which occur frequently in renal cell carcinomas, render these cancer cells resistant to ferroptosis, thereby uncovering a pro-oncogenic mechanism dependent on intracellular iron regulation.

## RESULTS

### IRP2-Clover reporter cells provide a sensitive and dynamic readout for intracellular iron levels

**Fig. 1.**
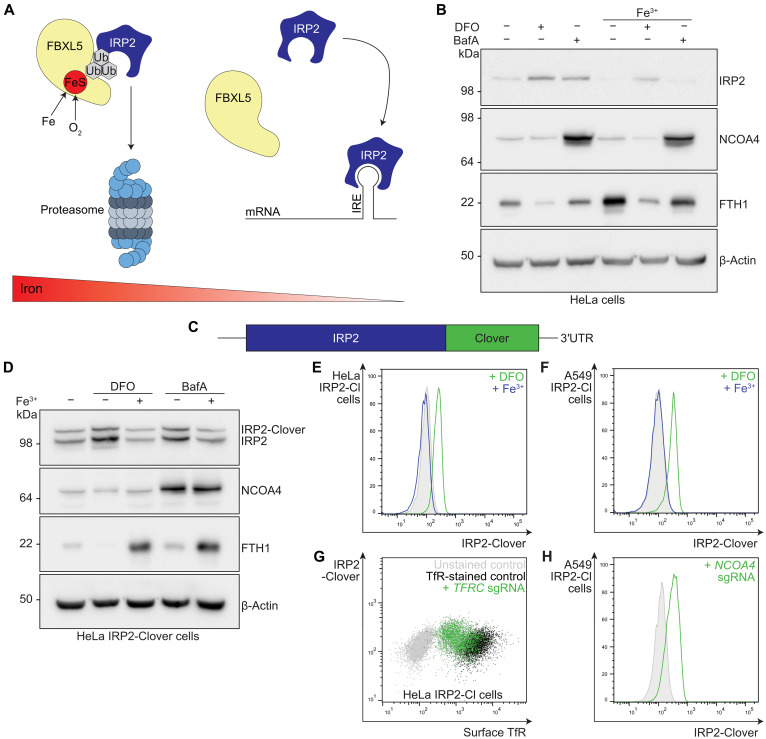
IRP2-Clover reporter cells provide a sensitive and dynamic readout for intracellular iron levels. (**A**) Under conditions of iron repletion, IRP2 is targeted for proteasomal degradation by FBXL5 in an oxygen-dependent manner, whereas when iron is deplete, IRP2 binds IREs to exert posttranscriptional control of cellular iron homeostasis. (**B**) HeLa cells were treated with DFO (100 μM, 24 hours) or BafA (10 nM, 24 hours) with or without ferric iron supplementation (FAC, 100 μM, 24 hours) and protein levels of IRP2, ferritin heavy chain (FTH1), ferritinophagy cargo receptor NCOA4, and β-actin assessed by immunoblot (*n* = 3). (**C**) Schematic of the IRP2-Clover reporter construct. (**D**) HeLa IRP2-Clover cells were treated with iron depletion by BafA (10 nM, 20 hours) or DFO (100 μM, 20 hours) with or without addition of iron. Cells were analyzed by immunoblot for IRP2, NCOA4, FTH1, and β-actin (*n* = 4). (**E**) HeLa IRP2-Clover cells were treated with iron depletion by DFO (200 μM, 20 hours) with or without addition of iron and analyzed by flow cytometry (*n* = 5). (**F**) A549 IRP2-Clover cells were treated with iron depletion by DFO (200 μM, 20 hours) with or without addition of iron and analyzed by flow cytometry (*n* = 3). (**G**) HeLa IRP2-Clover cells were transduced with sgRNA targeting *TFRC* with cell surface antibody staining for TfR and analysis by flow cytometry (*n* = 3). (**H**) Cells were transduced with sgRNA targeting *NCOA4* before analysis by flow cytometry (*n* = 3). IRP2-Cl, IRP2-Clover.

To map the landscape of intracellular iron metabolism, we first generated tools to measure iron flux. We focused on IRP2 as it provides the main cytosolic sensing arm of intracellular iron availability and IRP2 protein levels directly correspond to iron deficiency (*3*). Under conditions of iron repletion, IRP2 is ubiquitinated and targeted for proteasomal degradation by the FBXL5 Skp1-Cullin-F-box ubiquitin ligase complex in an iron-dependent manner (Fig. 1A) (*38*, *39*). Conversely, under conditions of iron depletion, IRP2 is stabilized and accumulates, binding IREs in the 5′ or 3′ untranslated regions (UTRs) of relevant mRNAs to exert posttranscriptional control of iron-relevant genes (*3*).

We used two strategies to determine the utility of IRP2 as a readout for intracellular iron availability. We first determined whether iron availability could be measured by IRP2 intracellular antibody staining and flow cytometry. HeLa cells treated with the iron-chelator desferrioxamine (DFO; 100 μM, 24 hours) showed an increase in IRP2 levels by both immunoblot and flow cytometry (Fig. 1B and fig. S1A). IRP2 accumulation was reversed by addition of exogenous ferric iron [ferric ammonium citrate (FAC); 100 μM, 24 hours]. In addition, inhibition of iron flux using the v-ATPase inhibitor, bafilomycin (BafA; 10 nM, 24 hours) resulted in IRP2 accumulation, which was reversible by addition of iron, confirming the utility of IRP2 as a robust readout of iron levels (Fig. 1B and fig. S1B).

Second, we developed HeLa and A549 clonal knock-in IRP2-Clover cell lines by endogenously tagging the C terminus of IRP2 with the fluorescent protein Clover (Fig. 1C and fig. S1C). Immunoblot and flow cytometry confirmed that IRP2-Clover accumulation occurred following iron depletion (iron chelation or v-ATPase inhibition) (Fig. 1, D to F, and fig. S1, D and E) (*40*, *41*), and ferric iron supplementation (FAC, 200 μM, 24 hours) reversed IRP2-Clover accumulation (Fig. 1, D to F, and fig. S1, D and E). To determine whether the IRP2-Clover cells would also respond to genetic manipulation of genes involved in iron regulation, we knocked out *TFRC* and *NCOA4* using single guide RNA (sgRNA) and observed that IRP2-Clover levels increased when either TfR uptake or ferritinophagy were prevented (Fig. 1, G and H, and fig. S1F).

### Genome-wide mutagenesis screens define the key regulators of intracellular iron metabolism

**Fig. 2.**
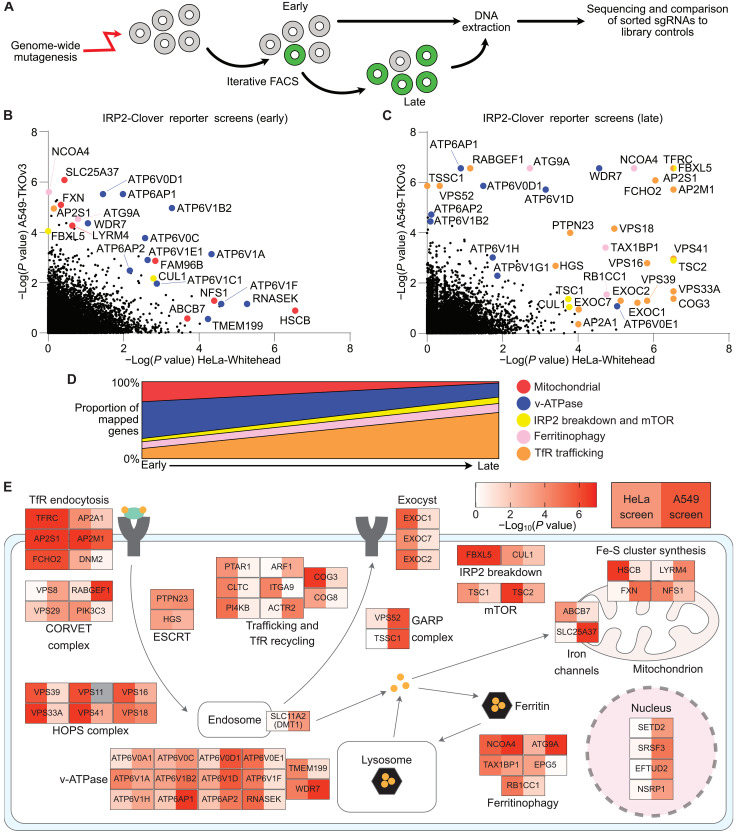
Genome-wide mutagenesis screens define the key regulators of intracellular iron metabolism. (**A**) HeLa or A549 IRP2-Clover cells expressing Cas9 were mutagenized with genome-wide sgRNA libraries, selected for lentiviral integration, and underwent FACS for Clover^HIGH^ cells after 8 days. Sorted cells were split between lysis for immediate DNA extraction and expansion for a second sort (days 16 to 18). DNA from phenotypically nonselected library cells was extracted for comparison, and cells were pooled before any selection event. (**B** and **C**) Bubble plots showing screen hits (genes overrepresented in sorted cells, as calculated by MAGeCK) identified in the A549 IRP2-Clover-TKOv3 screen (*y* axis) compared to the HeLa IRP2-Clover-Whitehead screen (*x* axis) at early (B, day 8) and late (C, day 16 or day 18, respectively) time points. Genes with related functions are highlighted. (**D**) Genes identified in either screen as top hits [−log(*P* value) >3] at early or late time points were manually annotated for function to assess changes over time, with genes of unknown function excluded (49/72 unknown at early time point and 44/104 unknown at late time point). (**E**) Across the HeLa IRP2-Clover-Whitehead and A549 IRP2-Clover-TKOv3 screens (both early and late time points), 150 unique genes were identified as registering a −log(*P* value) of >3 by MAGeCK analysis. Of these, 61 mapped to complexes and pathways with identifiable roles in cellular iron metabolism and 4 could be localized to the nucleus. For each gene, the color in the left box represents the highest −log(*P* value) of the two time points assessed for the HeLa IRP2-Clover screen and the color in the right box corresponds to the equivalent value for the A549 IRP2-Clover screen. IRP2-Cl, IRP2-Clover; MAGeCK, Model-based Analysis of Genome-wide CRISPR-Cas9 Knockout.

Having established the utility of IRP2 as a sensitive readout for cellular iron regulation, we used CRISPR-Cas9 mutagenesis screens to find the key genes and pathways regulating iron homeostasis. A549 or HeLa IRP2-Clover cells, stably expressing Cas9, were transduced with genome-wide sgRNA knockout (KO) libraries and underwent iterative fluorescence-activated cell sorting (FACS) to enrich for IRP2-Clover^HIGH^ levels, thus identifying genes that, when lost, promote IRP2 accumulation and by implication lead to reduced iron availability (Fig. 2A). Two sgRNA libraries were used to mitigate against differences in sgRNA design: The Whitehead library was transduced into HeLa IRP2-Clover cells, whereas the TKOv3 library was used in the A549 IRP2-Clover cells (*42*–*44*). We also compared the findings of the knock-in IRP2 reporters with an intracellular FACS screen, using antibody staining of endogenous IRP2 in HeLa cells (Whitehead or TKOv1 library) (fig. S2A) (*42*–*44*). For all screens, DNA was extracted at several time points postmutagenesis [days 7 to 8 (“early”) and days 14 to 17 (“late”)] to maximize the sensitivity of our approach and prevent the loss of genetic mutations that have more severe phenotypes (e.g., decreased cell growth or death).

All mutagenesis screens were analyzed using Model-based Analysis of Genome-wide CRISPR-Cas9 Knockout (MAGeCK), identifying key positive controls, including *FBXL5* and *TFRC* (Fig. 2B; fig. S2, B and C; and data S1), validating the approach. In addition, we identified a conserved group of genes potentially regulating iron levels (Fig. 2, B and C, and fig. S2, B and C). The IRP2-Clover reporter screens were more sensitive for detecting candidate genes compared to the antibody screens. In addition, the timing of flow cytometry sort postmutagenesis influenced the genes identified. For instance, genes annotated as mitochondrial regulators were found following the first round of FACS, whereas genes relating to endocytosis of the TfR were proportionally higher at later time points (Fig. 2D), likely reflecting the toxicity of perturbing mitochondrial function on cell growth.

We next generated a subpooled sgRNA library of the top hits identified in the screens (targeting 184 genes) to validate our findings in both HeLa and A549 IRP2-Clover cells (fig. S2D and data S2). By comparing the top hits across the primary and secondary screens, we identified a core set of genes that were reproducibly enriched for sgRNA (Fig. 2D). Whereas key regulatory genes (e.g., *FBXL5*, *NCOA4*, and *TFRC*) could be identified in all screens, other genes were identified at different time points postmutagenesis and in different cell lines, consistent with the temporal effects previously observed (Fig. 2, B to D, and fig. S2E) and suggesting some cell type–specific responses.

Lastly, we mapped the findings of the screens using gene ontology and known function. Gene ontology analysis confirmed enrichment of several pathways, including “synaptic vesicle cycle” as the most represented biological process, “endosome membrane” (containing the v-ATPase) as the most prominent cellular component, and “iron uptake and transport” as the top Reactome pathway (fig. S2F) (*45*–*47*). The top candidates in the A549 and HeLa IRP2-Clover screens were identified by using a negative logarithm of the *P* value for enrichment (calculated by MAGeCK) of ≥3 at either the early or late time point. This generated a list of 150 top-ranked genes (data S3). Of these, 61 genes could be mapped by function and grouped into pathways of cellular iron metabolism (Fig. 2E). TfR internalization and recycling dominated the top hits, with additional components such as the HOPS (homotypic fusion and protein sorting) and GARP (Golgi-associated retrograde protein) complexes identified relating to Tf recycling (*48*, *49*). The involvement of the v-ATPase in reducing ferric to ferrous iron was also apparent, as was the requirement for ferritinophagy (*21*, *40*). Less studied areas that were identified were the regulation of iron between the cytosol and mitochondria, the involvement of the exocyst complex, and the role of four additional nuclear localizing genes, including *SETD2* (mapped in Fig. 2E). Therefore, we chose to explore the relative contribution of the exocyst complex and mitochondria to iron metabolism and the role of SETD2.

### The exocyst complex and mitochondrial iron metabolism influence cellular iron flux

The exocyst is involved in trafficking of vesicles to the plasma membrane for secretion (*50*). Three genes encoding subunits of the exocyst (*EXOC1*, *EXOC2*, and *EXOC7*) were identified in our screens (Fig. 2, B and E), but the role of this complex in intracellular iron regulation was not clear. The exocyst may be required for TfR recycling in erythroblasts, but its role outside of erythropoiesis, where iron requirements are particularly high, has not been described (*51*). Therefore, we chose to knock down *EXOC1* by lentiviral short hairpin RNA (shRNA) transduction and measured the IRP2 response in A549 IRP2-Clover cells.

**Fig. 3.**
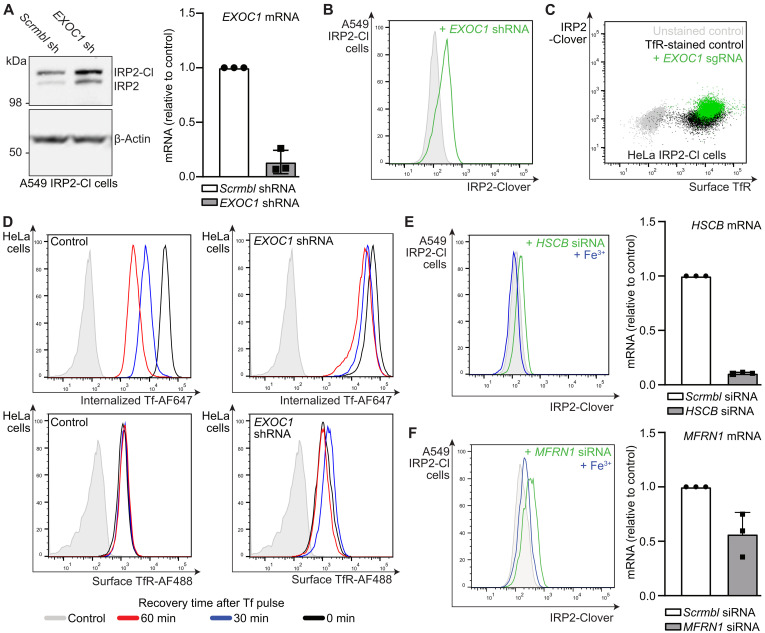
The exocyst complex, mitochondrial iron metabolism, and iron sulfur cluster assembly influence cellular iron flux. (**A**) A549 IRP2-Clover cells were transduced with shRNA targeting *EXOC1* and underwent immunoblot for IRP2 and β-actin. *EXOC1* depletion was confirmed by reverse transcription quantitative polymerase chain reaction (RT-qPCR) (*n* = 3). (**B**) A549 IRP2-Clover cells were transduced with shRNA targeting *EXOC1* and underwent flow cytometry for measurement of IRP2-Clover levels (*n* = 3). (**C**) HeLa IRP2-Clover cells were transduced with sgRNA targeting *EXOC1* and stained with antibody targeting surface TfR before analysis by flow cytometry (*n* = 2). (**D**) TfR uptake and recycling. HeLa cells with or without knockdown of *EXOC1* by shRNA were serum starved for 45 min, incubated with Tf-AF647 (5 μg/ml) for 5 min, washed with PBS, and recovered in serum-free medium for the indicated time periods. Internalized Tf-AF647 was assessed by flow cytometry. TfR surface levels were detected by flow cytometry (*n* = 3, note controls identical for both conditions). (**E**) A549 IRP2-Clover cells were transfected twice with siRNA targeting *HSCB* (48 and 24 hours before analysis by flow cytometry) ± ferric iron (FAC, 200 μM, 24 hours). Knockdown was confirmed by RT-qPCR (right) (*n* = 3). (**F**) A549 IRP2-Clover cells were transfected with siRNA targeting *MFRN1* ± ferric iron (FAC, 200 μM, 24 hours) before analysis by flow cytometry (left). Knockdown was confirmed by RT-qPCR (right) (*n* = 3). IRP2-Cl, IRP2-Clover.

Both endogenous IRP2 and IRP2-Clover levels increased following EXOC1 depletion (Fig. 3A). Similar findings were identified using shRNA targeting *EXOC1* in A549 and HeLa IRP2-Clover reporter cells (Fig. 3B and fig. S3A). However, although increased IRP2 levels suggested perturbation of iron flux when the exocyst was depleted, we did not observe any change in steady-state levels of cell surface TfR after KO of *EXOC1* in either HeLa or A549 IRP2-Clover cells (Fig. 3C and fig. S3A). Therefore, we used a Tf uptake and recycling assay with fluorescently labeled Tf (Tf-AF647) to establish how the exocyst may be implicated in intracellular iron regulation (Fig. 3D). HeLa cells were pulsed with Tf-AF647 for 5 min, and labeled Tf and cell surface TfR measured after 30 or 60 min. Tf-AF647 levels reduced at 30 and 60 min in control HeLa cells, consistent with rapid recycling of Tf to the plasma membrane (Fig. 3D). However, high levels of Tf-A647 were retained in EXOC1-depleted cells, indicating that TfR recycling was impaired (Fig. 3D). TfR levels also transiently increased at the cell surface in pulsed cells which had EXOC1 loss (Fig. 3D). Therefore, loss of the exocyst complex results in impaired iron flux due to defective TfR recycling, even in cells without enhanced iron requirements.

The identification of genes involved in mitochondrial iron metabolism suggested that the mitochondria themselves can affect cytosolic iron flux. Related genes identified in our screens included the mitochondrial iron sulfur (Fe-S) cluster chaperone *HSCB* and the mitochondrial iron importer *SLC25A37* [Mitoferrin-1 (*MFRN1*)]. Depleting HSCB using small interfering RNA (siRNA) resulted in an increase in IRP2-Clover levels, which returned to baseline when we supplemented the media with excess iron (Fig. 3E), thus confirming that normal intracellular iron flux was impaired and supporting a previous study on this chaperone (*52*). Depleting the mitochondrial iron import channel Mitoferrin-1 using siRNA also confirmed an iron-dependent increase in IRP2-Clover levels (Fig. 3F). We therefore assessed whether *SLC25A38* (Mitoferrin-2, *MFRN2*) could provide an alternative route for mitochondrial iron entry when MFRN1 was depleted; however *MFRN2* expression was not altered (fig. S3B). Therefore, both impaired Fe-S synthesis or reduced mitochondrial iron levels can result in accumulation of IRP2, potentially driving increased cytosolic iron levels as a compensatory response.

### Loss of the histone methylase SETD2 results in an iron-dependent IRP2 response

**Fig. 4.**
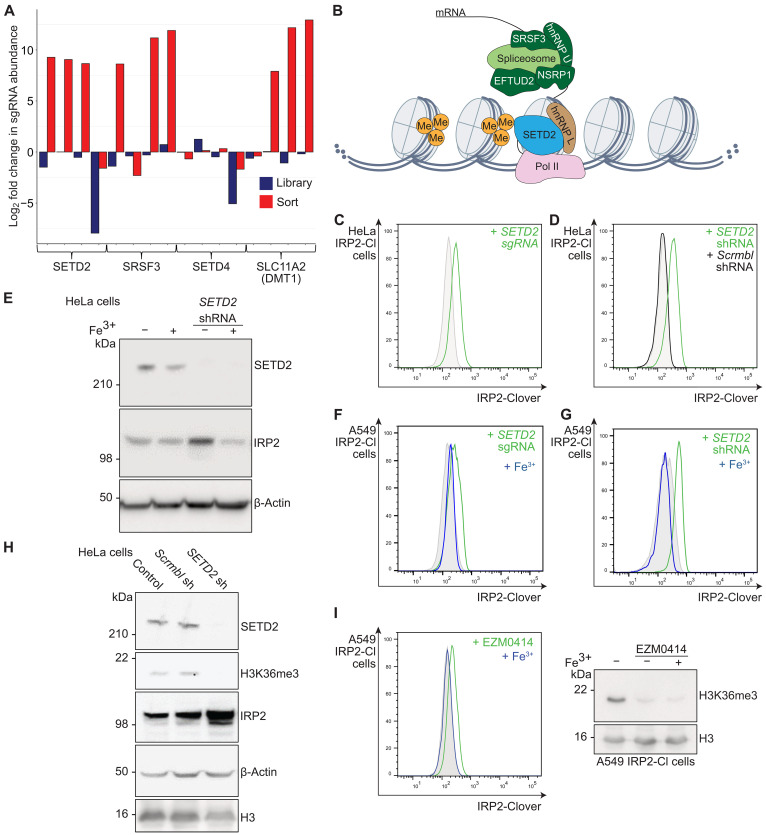
SETD2-mediated IRP2 accumulation is dependent on enzymatic activity. (**A**) Bar chart detailing the log_2_(fold change) in individual sgRNA counts for *SETD2* in the A549 IRP2-Clover CRISPR screen, as well as an associated splicing regulator (*SRSF3*). *DMT1* was included as a positive control, and *SETD4* was included as a negative control. (**B**) Schematic illustrating the canonical role of SETD2 in H3K36me3 and regulation of splicing. Spliceosome components enriched for sgRNA in the screens are shown in dark green. (**C** and **D**) HeLa IRP2-Clover cells were transduced with sgRNA targeting *SETD2* (C) (*n* = 4) or shRNA targeting *SETD2* with a shRNA scrambled control (D) (representative of at least three biological replicates). IRP2-Clover fluorescence was measured by flow cytometry. (**E**) HeLa cells were transduced with shRNA targeting *SETD2* and treated with or without ferric iron supplementation (FAC, 200 μM) for 24 hours before analysis by immunoblot (*n* = 4). (**F** and **G**) A549 IRP2-Clover cells were transduced with sgRNA (*n* = 3) or shRNA (*n* = 4) targeting *SETD2* and treated with or without ferric iron supplementation (FAC, 200 μM, 24 hours) before analysis by flow cytometry. (**H**) HeLa cells were transduced with shRNA targeting SETD2, and cells were lysed before analysis by immunoblot for levels of SETD2, H3K36me3, IRP2, β-actin, and H3 (*n* = 4). (**I**) A549 IRP2-Clover cells were treated with EZM0414 (200 nM, 48 hours), with or without ferric iron supplementation (FAC, 200 μM, 24 hours) before analysis by flow cytometry for IRP2-Clover levels (left) and immunoblot for H3K36me3 and H3 (right) (*n* = 3). IRP2-Cl, IRP2-Clover.

Cellular iron homeostasis is not primarily regulated by transcription. Therefore, the identification of *SETD2*, a methyltransferase responsible for adding the H3 lysine 36 trimethylation (H3K36me3) mark (*53*, *54*), as a hit within the genetic screens was intriguing (Fig. 4A and fig. S4A). SETD2 also associates with the spliceosome (*55*), and several spliceosome genes were identified in our IRP2-Clover reporter screens (*SRSF3*, *NSRP1*, *EFTUD2*, and *HNRNPU*), suggesting a possible transcriptional role of a SETD2 complex in iron metabolism (Fig. 4, A and B). Therefore, we examined whether SETD2 loss altered iron flux and whether this was dependent on its catalytic activity.

We first validated that SETD2 depletion increased IRP2 levels using sgRNA or shRNA-mediated depletion in IRP2-Clover reporter cells (Fig. 4, C and D). A similar increase in untagged endogenous IRP2 following SETD2 depletion was seen in HeLa cells (Fig. 4E). We also observed IRP2 accumulation in HAP1, 786-O, and MCF7 cells lines when SETD2 was depleted, confirming the generalizability of our findings (fig. S4, B to D). IRP2 accumulation following SETD2 loss was dependent on iron flux as ferric iron (FAC, 200 μM, 24 hours) reduced IRP2 levels in both HeLa cells and A549 IRP2-Clover reporter cells (Fig. 4, E to G). Therefore, depletion of SETD2 results in accumulation of IRP2, in an iron-responsive manner.

As the main function of SETD2 relates to its methyltransferase activity (*53*, *54*), we examined whether SETD2 inhibition altered iron flux. SETD2 shRNA-mediated depletion or inhibition with EZM0414 (200 nM, 48 hours) (*56*) decreased H3K36me3 levels (Fig. 4, H and I), as expected. IRP2-Clover levels also increased following SETD2 inhibition in an iron-dependent manner, similarly to SETD2 depletion (Fig. 4I). Given that iron depletion has been associated with increased H3K36me3 (*33*), we next examined whether SETD2 could be acting as an iron sensor, but levels of SETD2 itself were not altered by perturbations in iron availability (fig. S5A). Depletion of CD44, an iron importer that is regulated by H3K36me3 (*31*), also did not recapitulate an IRP2^HIGH^ phenotype (fig. S5B).

Last, we considered whether noncanonical roles of SETD2 may contribute to the perturbation in iron homeostasis as non-histone methylation by SETD2 has been reported on microtubules and actin, where it alters polymerization (*57*, *58*). However, the subcellular localization of SETD2 argued against a noncanonical role of SETD2 as both immunofluorescence and subcellular fractionation assays showed that SETD2 was bound primarily to chromatin in HeLa cells (fig. S5, C and D). In addition, we did not observe IRP2 accumulation with inhibition of microtubule polymerization (CK-869, 200 nM, 24 hours) or reversal of IRP2 levels with the actin polymerization promoter jasplakinolide (100 nM, 24 hours) (fig. S5E). Together, these findings support SETD2 acting via its canonical H3K36me3 function to drive IRP2 accumulation.

### SETD2 deficiency results in defective ferritinophagy

**Fig. 5.**
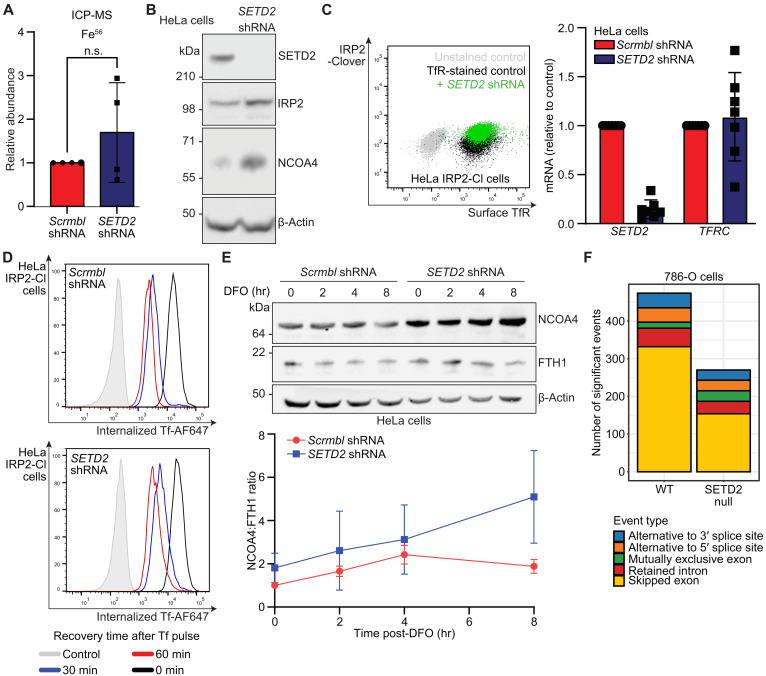
Loss of SETD2 results in increased NCOA4 and altered ferritinophagy. (**A**) HeLa cells were transduced with shRNA targeting *SETD2* or a scrambled control. Cells were lysed in nitric acid before analysis by ICP-MS. Duplicate cell cultures were counted and measurements normalized to cell number (*n* = 4 in technical triplicate; *P* = 0.31, paired *t* test). n.s., not significant. (**B**) HeLa cells were transduced with shRNA targeting *SETD2* and protein levels of SETD2, IRP2, NCOA4, and β-actin measured by immunoblot (representative of at least three biological replicates). (**C**) HeLa and HeLa IRP2-Clover cells were transduced with shRNA targeting *SETD2* and underwent flow cytometry for surface TfR and IRP2-Clover (*n* = 3). Transcript levels of *SETD2* and *TFRC* were measured by RT-qPCR (*n* = 7). (**D**) TfR uptake and recycling. SETD2 was depleted in HeLa IRP2-Clover cells using shRNA, along with a shRNA scrambled control. Cells were serum starved for 5 min, incubated with Tf-AF647, and recovered in serum-free medium for the indicated time points. Internalized Tf-AF647 was assessed by flow cytometry (*n* = 3). (**E**) Control or SETD2-depleted HeLa cells (shRNA) were treated with DFO (100 μM) for 0 to 8 hours and levels of NCOA4, FTH1, and β-actin measured by immunoblot (*n* = 3). Immunoblots were quantified and normalized to loading and 0-hour time points before calculation of the NCOA4:FTH1 ratio. hr, hours. (**F**) Publicly available RNA-seq data from wild-type and SETD2 stable KO (SETD2 null) 786-O cells (GSE150609) were analyzed using rMATS, and splicing events were quantified. IRP2-Cl, IRP2-Clover.

To understand how SETD2 may influence iron flux, we examined which aspect of intracellular iron metabolism was impaired when SETD2 was depleted. Although total cell iron levels remained constant (Fig. 5A), a notable finding was the accumulation of the ferritinophagy cargo receptor, NCOA4, in SETD2-deficient cells (Fig. 5B). Perturbations in ferritinophagy may arise as a compensatory mechanism for reduced iron import (*59*–*63*), and as SETD2 modulates transcription, we first considered whether the TfR pathway may be impaired following SETD2 loss. However, *TFRC* mRNA levels were not altered following SETD2 depletion (Fig. 5C and fig. S6A). Moreover, cell surface levels of TfR remained similar to those seen in SETD2-replete HeLa or A549 IRP2-Clover cells (Fig. 5C and fig. S6B). Tf uptake and recycling was also not altered by SETD2 loss (Fig. 5D). Therefore, as the Tf pathway could not account for the alterations in iron flux following SETD2 depletion, we focused on ferritin breakdown or ferritinophagy.

NCOA4 accumulation is typically observed when ferritinophagy is impaired as NCOA4 is codegraded with ferritin within the lysosome (*21*, *40*), but NCOA4 levels may also relate to variations in Fe-S cluster binding (*63*–*65*). Therefore, to assess whether SETD2 loss impaired ferritinophagy, we measured the kinetics of NCOA4 stability in HeLa cells. NCOA4 levels were higher at baseline in SETD2-depleted cells and increased over time after iron depletion with DFO (100 μM, 0 to 8 hours) (Fig. 5E). In addition, FTH1 levels were relatively preserved after iron depletion, with a persistent increase in the NCOA4:FTH1 protein ratio, consistent with SETD2 loss impairing ferritinophagy (Fig. 5E). To test the involvement of Fe-S cluster binding in NCOA4 levels, we depleted a known Fe-S cluster biosynthesis gene (*ISCA2*) and measured NCOA4 stability via a cycloheximide (CHX) chase. We confirmed that ISCA2 depletion impaired Fe-S synthesis as we detected a decrease in lipoylation—a posttranslational modification dependent on the enzymatic activity of Fe-S containing lipoic acid synthase (fig. S6C) (*66*). No change in NCOA4 levels was observed following ISCA2 depletion. In contrast, NCOA4 levels increased with SETD2 inhibition (fig. S6C). Fe-S binding was therefore unlikely to account for increased NCOA4 following SETD2 loss.

To further delineate the involvement of SETD2 in ferritinophagy, we selectively depleted autophagy adaptors and examined whether they abrogated the effect of SETD2 on NCOA4. KO of *ATG9A*, an autophagy component required for ferritinophagy (*67*), increased NCOA4 levels as expected (fig. S6D). SETD2 depletion in *ATG9A* null cells did not alter NCOA4 further (fig. S6D), consistent with SETD2 loss impairing ferritinophagy in an ATG9A-dependent manner. *ATG16* null cells, which have impaired phagophore expansion but not ferritinophagy (*67*–*69*), showed no change in baseline NCOA4 levels, whereas SETD2 loss did still result in increased NCOA4 abundance (fig. S6D). Overall, these findings are consistent with SETD2 regulating ferritin flux via ferritinophagy.

Why a chromatin-associated methyltransferase involved in transcriptional activation, fidelity, and splicing should regulate ferritinophagy was not clear. Therefore, we examined whether SETD2 loss altered histone methylation, transcription, or splicing of genes relating to iron metabolism using available RNA sequencing (RNA-seq) and chromatin immunoprecipitation sequencing (ChIP-seq) data (*70*). We first generated a list of iron-relevant genes using gene ontology designations (data S2) and examined whether SETD2 loss altered transcription of these key iron regulatory genes or ferritinophagy adaptors. H3K36me3 levels were globally reduced following SETD2 depletion, but no specific differences were observed at genes involved in iron homeostasis (fig. S7A). The mRNA levels of these iron regulatory genes were also not altered by SETD2 loss (fig. S7, B and C).

We next considered whether SETD2 was required for the transcriptional fidelity of IREs and analyzed both H3K36me3 levels and transcript levels of known IRE-containing genes using ChIP-seq and RNA-seq data from SETD2-depleted HepG2 cells. No major differences in H3K36me3 levels (fig. S8A) or transcript levels were observed (fig. S8B). We also examined whether SETD2 loss altered transcript levels of the *TFRC* 3′ UTR which contains IREs, as, if these were altered by SETD2 loss, iron uptake would be reduced. However, no changes were observed in this section of the *TFRC* transcript (fig. S8C). Therefore, it was unlikely that SETD2 deficiency was exerting effects on iron homeostasis directly through IRE-containing proteins. Instead, it was possible that SETD2 was required for correct splicing of ferritinophagy-related genes. Consistent with this notion, SETD2 loss resulted in a general reduction in splicing events, including a pronounced reduction in exon skipping (*71*), and perturbed splicing events (Fig. 5F). Isoform analysis of *NCOA4* identified differential expression when SETD2 was depleted (fig. S9A), along with *MFRN1* and *TFRC* (fig. S9, B and C), but not other genes related to iron metabolism (fig. S9, D to H). Overall, these findings indicate that genes involved in iron metabolism, including NCOA4, are susceptible to aberrant splicing when SETD2 is depleted or inhibited.

### Loss of SETD2 drives resistance to ferroptosis in cancer cells

**Fig. 6.**
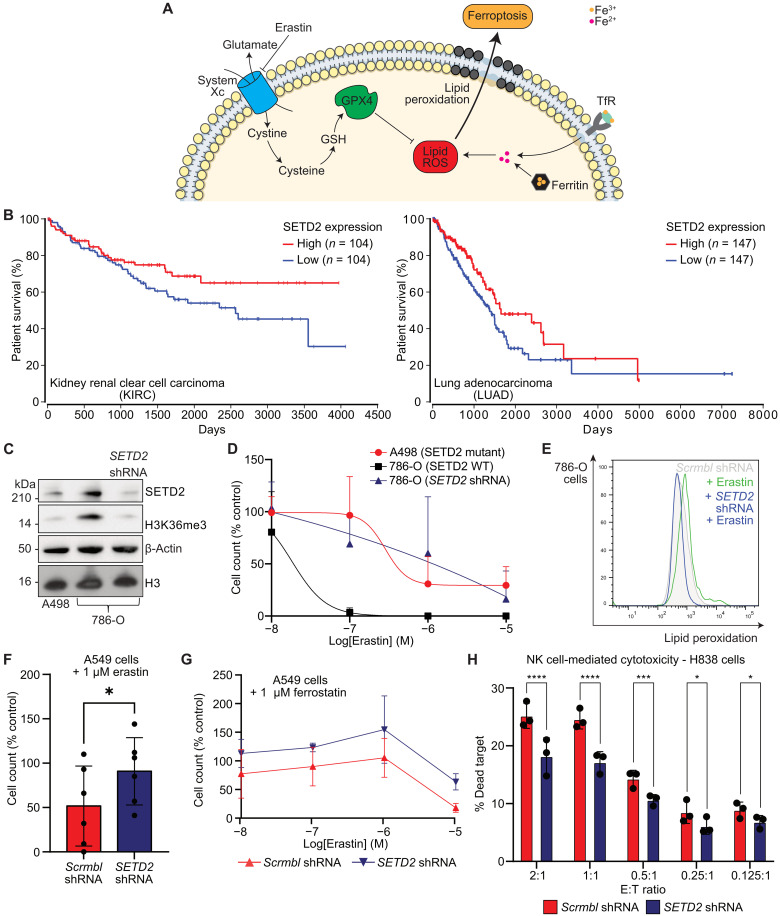
SETD2 depletion correlates with cancer cell survival and resistance to ferroptosis. (**A**) Schematic describing the key pathways in ferroptosis [membrane icon from Servier (https://smart.servier.com/), CC BY 3.0 Unported, https://creativecommons.org/licenses/by/3.0/deed.en]. ROS, reactive oxygen species. (**B**) Kaplan-Meier survival analysis for ccRCC and lung adenocarcinoma comparing survival of patients with tumors with high (top 30%) and low (bottom 30%) *SETD2* expression levels. Curves generated using OncoLnc, data from The Cancer Genome Atlas, log-rank *P* value = 0.02 (KIRC) and 0.04 (LUAD). (**C**) 786-O cells ± *SETD2* knockdown were analyzed by immunoblot for levels of SETD2, H3K36me3, β-actin, and H3 in comparison to 786-O wild-type and A498 wild-type cells (*n* = 3). (**D**) A498 cells and 786-O cells were treated with increasing concentrations of erastin and live cells counted after 24 hours. 786-O cells were transduced with shRNA targeting *SETD2* and the assay repeated (*n* = 5, as the percentage of untreated cells). (**E**) 786-O cells ± *SETD2* knockdown were treated with erastin (1 μM, 24 hours) and stained with BODIPY C11 (5 μM, 35 min) before analysis by flow cytometry (*n* = 3). (**F**) A549 cells ± *SETD2* knockdown were treated with erastin (1 μM, 48 hours) and live cells counted (*n* = 5, as the percentage of untreated cells; *P* = 0.03, paired *t* test). (**G**) A549 cells ± *SETD2* knockdown were treated with erastin (10 nM to 10 μM, 48 hours) and ferrostatin (1 μM, 48 hours) and live cells counted (*n* = 3, as the percentage of untreated cells). (**H**) H838 cells ± *SETD2* knockdown were incubated with primary ex vivo expanded human NK cells for 4 hours at a range of effector:target (E:T) cell ratios, with both cell groups labeled (CFSE or Tag-IT Violet). Proportion of dead target cells was measured by Fixable Viability Dye eFluor 780 staining and flow cytometry (*n* = 3 biological replicates; ****P < 0.0001, ****P < 0.0001, ***P = 0.0007, *P = 0.0147, *P = 0.0358, two-way repeated measures ANOVA with a post hoc test for each E:T ratio).

Pharmacological or genetic disruption of iron availability can reduce activation of ferroptosis (Fig. 6A) (*22*, *72*). Given that inducing ferroptosis is a potential therapeutic strategy for targeting tumors and that *SETD2* is frequently mutated in cancer, we asked whether SETD2 loss may render some cancers resistant to ferroptosis. We focused on clear cell renal cell carcinoma (ccRCC), as a cancer where *SETD2* mutations contribute to tumorigenesis (*73*), and lung adenocarcinoma, where *SETD2* mutations are also known to occur (*74*). In both these cancer types, high SETD2 expression correlates with worse patient outcomes (Fig. 6B) (*75*).

We first tested whether known *SETD2* mutations altered cell death by ferroptosis in ccRCC, using A498 cells, which are SETD2 deficient due to loss of one allele on the short arm of chromosome 3 (3p) and a frameshift mutation in the second allele (A498 *SETD2^−/−^*), and 786-O ccRCCs, which encode a functional *SETD2* (Fig. 6C). We treated these cells with increasing concentrations of the ferroptosis inducer, erastin, and measured cell survival after 48 hours (Fig. 6D). A498 *SETD2*^−/−^ cells were resistant to erastin treatment compared to the 786-O *SETD2^+/+^* cells [log median inhibitory concentration (IC_50_): −6.5 versus −10.4]. To confirm that this difference in ferroptosis susceptibility was due to SETD2 levels, we depleted SETD2 in 786-O cells. Now, we observed a similar resistance to erastin treatment in 786-O *SETD2^−/−^* as A498 *SETD2^−/−^* cells (Fig. 6D). We also observed decreased lipid peroxidation by BODIPY-C11 staining in 786-O *SETD2^−/−^* cells (log IC_50_: −5.8) (Fig. 6E), consistent with decreased ferroptosis. To confirm that the effects of SETD2 depletion were not just confined to ccRCC, we examined the effects of SETD2 depletion A549 lung adenocarcinoma cells. Again, SETD2 depletion resulted in resistance to ferroptosis (Fig. 6F), and cotreatment with the ferroptosis inhibitor ferrostatin (1 μM) was synergistic with SETD2 depletion (Fig. 6G).

Inducing ferroptosis in cancers is being pursued for therapeutic effect, but ferroptosis also contributes to cancer immunosurveillance (*26*). Therefore, to explore whether SETD2 may enhance cancer cell killing by immune cells, we focused on NK cells as enhanced cancer cell killing has been reported with ferroptosis agents (*30*). We isolated NK cells from human donors and first established which cancer cells were susceptible to NK killing. Of the kidney and lung adenocarcinoma lines tested, H838 lung adenocarcinoma cells proved to be a cell type susceptible to NK killing, and this susceptibility is in keeping with a prior work (*76*). We then measured NK-mediated cytotoxicity against SETD2-proficient or SETD2-deficient H838 cells. SETD2 depletion reduced NK killing of H838 cells, with the most marked changes observed at higher effector:target ratios (Fig. 6H). Therefore, SETD2 depletion both enhances resistance to ferroptosis induction and may contribute to how some cancers evade immunosurveillance.

## DISCUSSION

How iron flux is controlled in cells has been difficult to address. In part, this relates to technical limitations in measuring free iron or estimating the LIP. Our generation of an endogenous IRP2 fluorescent reporter allows measurement of perturbations in iron flux, and the genetic screens using this reporter provide a comprehensive annotation of the key processes involved. TfR uptake and recycling dominate these screens, as anticipated, but we also identified an unexpected role for mitochondrial signaling back to the cytosol and uncovered a nuclear component in controlling iron flux, via the SETD2 histone methyltransferase.

Our data indicate that ferritinophagy is particularly susceptible to SETD2 loss, but how precisely this relates to the catalytic activity of SETD2 remains to be fully determined. Aberrant splicing may explain the observed uncoupling of ferritin breakdown, where NCOA4 levels accumulate along with a relative cellular iron deficiency. Other genes involved in cellular iron homeostasis were also susceptible to aberrant splicing, including *MFRN1* and *TFRC*, but protein levels of TfR were not altered by SETD2 loss, whereas NCOA4 accumulated. Why ferritinophagy and NCOA4 should be more susceptible than other cellular processes to aberrant splicing is unclear, but *SETD2* mis-splicing has been reported for other autophagy genes unrelated to ferritinophagy (*77*–*79*).

Our results regarding the susceptibility to ferroptosis of ccRCC cell lines highlight why *SETD2* mutations may be advantageous in the evolution of the cancer. They are also in line with work profiling the sensitivity of ccRCC lines to erastin-induced cell death (*80*), where A498 *SETD2^−/−^* cells were more resistant to erastin than 786-O *SETD2^+/+^* cells. The effect of *SETD2* mutations on ferroptosis has broader disease implications as *SETD2* mutations occur in ~10% of lung adenocarcinomas (*74*). However, determining the downstream consequences for *SETD2* mutations in each cancer type will be important as death by ferroptosis will depend on other pathways involved. For example, the Fe-S cluster biogenesis enzyme NFS1 is highly expressed in lung adenocarcinoma, where it protects against the induction of ferroptosis by oxidative damage resulting from high oxygen (*27*). Conversely, epidermal growth factor receptor (EGFR) mutated non–small cell lung cancers can be particularly sensitive to the induction of ferroptosis after cystine depletion (*29*). Therefore, it will be of interest in future work to understand the relationship between SETD2 loss and other somatic mutations involved in lung adenocarcinoma development.

Our genetic screens provide a global perspective of intracellular iron metabolism, but there are some limitations. Genes identified in the screens were not consistent across all time points or cell types. Although this is not unexpected, it does suggest that different cell types can have an altered reliance on iron uptake versus ferritin breakdown. We would caution against excluding genes not identified in our screens from further studies, based only on the cutoff values used here, as we cannot exclude that some genes that were not enriched may still be important for iron regulation in different cell types. Last, SETD2 loss or inhibition may protect against ferroptosis by additional mechanisms to iron depletion alone, and regulation of mediators, such as cystine, glutamate, and GPX4, will require consideration in future studies. However, our work provides insights into how *SETD2* mutations may contribute to cancer evolution through resistance to ferroptosis and NK cell evasion, and this will be important to consider in the context of ferroptosis inducers as cancer treatments.

## MATERIALS AND METHODS

### Cell culture

HeLa, A549, human embryonic kidney (HEK) 293T, MCF7, HepG2, and A498 cells were maintained in Dulbecco’s modified Eagle’s medium (DMEM; Sigma-Aldrich, D6429) supplemented with 10% fetal bovine serum (FBS; Sigma-Aldrich, F0392). 786-O and H838 cells were maintained in RPMI (Sigma-Aldrich, R8758) supplemented with 10% FBS. HAP1 cells were maintained in Iscove’s modified Dulbecco’s medium (IMDM; Gibco, 31980030) supplemented with 10% FBS. Iron levels in media supplemented with FBS are ~5 μM (*81*). Culture medium was not supplemented with penicillin-streptomycin outside of cell sort recovery. All cells were maintained in a 5% CO_2_ incubator at 37°C. HeLa ATG9 null, HeLa ATG16 null, and their paired control cells were gifts from D. Rubinsztein (CIMR, Cambridge). A498 cells were a gift from P. Schraml (Zurich). Mycoplasma testing was performed on a monthly basis (MycoAlert, Lonza LT07-318). All cells were authenticated (Eurofins).

### Immunoblotting

Cells were lysed in an SDS lysis buffer [2% SDS, 50 mM Tris (pH 7.4), 150 mM NaCl, 10% glycerol, and 1:200 DENARASE] on ice. Electrophoresis and transfer were performed before incubation with primary and secondary antibodies (table S1). Immunoblots were developed using Pierce ECL or West Pico Plus before imaging (Thermo Fisher Scientific, 32209 and 34577) via iBright (Thermo Fisher Scientific) or ChemiDoc MP Imaging System (Bio-Rad, 17001402). Immunoblots are representative of at least three independent experiments unless otherwise noted.

### Subcellular fractionation

Cells were lysed in Buffer A [10 mM Hepes, 1.5 mM MgCl_2_, 10 mM KCl, 0.5 mM dithiothreitol (DTT), and EDTA-free protease cocktail tablet (Roche)] with 0.1% NP-40 and incubated [10 min, shaking, room temperature and pressure (RTP)]. 50 μl was taken as a total cell lysate control and the remainder underwent centrifugation (1400*g*, 4 min, 4°C), followed by collection of the supernatant (cytosolic fraction). The nuclear pellet was resuspended in Buffer B (20 mM Hepes, 1.5 mM MgCl_2_, 300 mM NaCl, 0.5 mM DTT, 25% glycerol, 0.2 mM EDTA, and EDTA-free protease inhibitor cocktail tablet) (10 min, on ice) and centrifuged (1700*g*, 4 min, 4°C). The supernatant (nucleosolic fraction) was collected, and the remaining pellet was lysed in SDS with DENARASE (chromatin fraction).

### Immunofluorescence

Cells were seeded on FBS-coated coverslips in 24-well plates. After 24 hours, cells were washed [Dulbecco’s phosphate-buffered saline (DPBS), Merck, D8662], fixed in 4% paraformaldehyde (PFA), permeabilized with 0.1% Triton (10 min), blocked in 4% FBS in DPBS (1 hour, RTP), and incubated with primary antibody in 40 μl of blocking buffer (overnight, 4°C) (table S1). Coverslips were returned to the plate and washed before incubation with secondary antibody in 200 μl of blocking buffer for (1.5 hours, RTP). Coverslips were mounted on slides with a glycerol-based mount containing 4′,6-diamidino-2-phenylindole (DAPI) (CitiFluor AF1 + DAPI, Electron Microscopy Sciences, D17970) and kept at 4°C until analysis (Zeiss, LSM 980). Images were processed using ImageJ.

### Flow cytometry

Cells were resuspended in PBS before flow cytometry (BD Fortessa; software: FACSDiva 8.0). Flow cytometry plots are representative of at least three independent experiments unless otherwise noted. For intracellular flow cytometry, cells were washed with PBS and fixed in 90% ice-cold methanol for 30 min. Primary antibody was then added to these fixed, permeabilized cells for 45 min (table S1). Secondary fluorescent antibody was added and incubated in darkness at room temperature for 30 min (table S1).

### TfR recycling assay

Cells were serum starved for 45 min, incubated with Tf-AF647 (5 μg/ml) for 5 min, washed with PBS, and recovered in serum-free medium at indicated time points. Internalized Tf-AF647 and surface TfR levels were assessed by flow cytometry.

### CHX chase assay

Cells were treated with CHX (10 ng/μl) for the indicated times before analysis by immunoblot.

### Molecular biology

The lentiviral sgRNA expression vector pKLVU6gRNA(BbsI)-PGKpuro2ABFP was a gift from K. Yusa (Addgene, #50946) (*82*). The lentiviral shRNA expression vector pc-SIREN was a gift from P. Lehner (CITIID). Polymerase chain reaction (PCR) amplification was typically performed in 50-μl reactions using Phusion High Fidelity DNA polymerase (NEB, M0530) per the manufacturer’s protocol. Restriction digests were performed using the relevant enzymes per the manufacturer’s instructions, typically FastDigest enzymes (Thermo Fisher Scientific). DNA was purified by agarose gel electrophoresis (supplemented with SYBR Safe, Thermo Fisher Scientific) and bands extracted and recovered using a Zymoclean Gel DNA Recovery kit per the manufacturer’s instructions (Zymo Research). Bacterial transformation was performed by adding plasmid to competent *Escherichia coli* on ice for 20 min, before heat shock at 42°C for 45 s, followed by recovery in super optimal broth and spreading on LB agar plates containing the relevant antibiotic and overnight incubation (37°C). Individual colonies were selected and further incubated at 37°C in LB supplemented with antibiotics before plasmids were purified using either the QIAGEN QIAprep Spin Miniprep Kit or Maxi Kit per the manufacturer’s instructions. Successful cloning was confirmed by commercial sequencing (GENEWIZ, Azenta Life Sciences).

Knock-in reporter cell lines were generated by constructing a pDonor-IRP2-Clover plasmid comprising IRP2 5′ and 3′ homology arms, a Clover-Puromycin resistance vector fragment, and a kanamycin resistance vector fragment with Gibson assembly primers listed in table S2. This construct was transfected into cells with alongside sgRNAs targeting the C terminus or 3′UTR of IRP2 in a transient Cas9 expression vector. Cells were selected by puromycin, and then the puromycin resistance cassette was removed by treatment with Cre recombinase or a self-excising retroviral vector encoding Cre recombinase (pHR-MMPCreGFP, a gift from J. Jonkers, Universiteit Leiden) (*83*). Single-cell clones were then isolated, and IRP2-Clover integration was confirmed by PCR sequencing.

### Lentiviral production and transduction

HEK293T cells were transfected at ~70% confluence using Fugene 6 (Promega UK E2692) at a ratio of 6 μl of transfection reagent to 2 μg of DNA. DNA was composed of pCMV-dR8.91 (gag/pol), the relevant expression plasmid, and pMD.G [vesicular stomatitis virus glycoprotein (VSVG)] in a 2:3:4 ratio. The supernatant was collected at 48 hours through a 0.45-μm filter. Transduction was performed by addition of a minimum of 200 μl of the supernatant to cells in media, subsequent incubation at 37°C, and addition of further medium after 3 hours. Cells were selected after a minimum of 27 hours with antibiotic selection applied for 48 hours. Lentiviral packaging vectors pCMV-dR8.91 (gag/pol) and pMD.G (VSVG) were gifts from P. Lehner (CITIID).

### Knockdown

Sequences for shRNAs were generated via the Broad Institute’s RNA interference Platform library. Oligos were designed with a TTCAAGAGA hairpin sequence, purchased commercially, and cloned into a pc-SIREN backbone. Plasmids were packaged into lentivirus and transduced into cells before assay.

Knockdown by siRNA was adapted from a protocol by Uhrigshardt *et al.* (*84*) with consecutive transfections 24 and 48 hours before assay. Sequences were generated by Dharmacon (*MFRN1*) and Uhrigshardt *et al.* (*84*) (*HSCB*) and purchased from Eurofins (scrambled control and *MFRN1*) and IDT (*HSCB*) (table S3).

### Reverse transcription quantitative polymerase chain reaction

Total RNA was extracted using the PureLink RNA Mini Kit (Thermo Fisher Scientific, 12183018A) according to the manufacturer’s instructions. RNA was reverse transcribed using ProtoScript II Reverse Transcriptase (NEB) according to their standard protocol (NEB, #M0368). Template cDNA (40 ng) was amplified using SYBR Green PCR Master Mix (Thermo Fisher Scientific, 4309155) and QuantStudio 7 Real-Time PCR System (Thermo Fisher Scientific). Transcript levels of genes were normalized to β-actin. Primer sequences were generated by PrimerBank or obtained from published protocols (table S4) (*85*, *86*).

### CRISPR-Cas9 knockout

For stable expression of Cas9, cells were transduced with pHRSIN-FLAG-NLS-CAS9-NLS-pGK-Hygro (a gift from R. Timms and P. Lehner) or Lenti Cas9-T2A-Blast (a gift from J. Moffat, Addgene, #73310) (*43*). Gene-specific sgRNA sequences (table S5) were generated from genome-wide CRISPR-Cas9 libraries, eCRISP, or Vienna BioCentre (*87*, *88*) and cloned into a pKLV-U6sgRNApGKPuro-2A-BFP backbone.

### CRISPR-Cas9 forward genetic screens

The Human Two Plasmid Activity-Optimized CRISPR “Whitehead” Knockout Library was a gift from D. Sabatini and E. Lander (Addgene, #1000000095) (*42*). The Toronto human knockout pooled library (TKO) was a gift from J. Moffat (Addgene, #1000000069) (*43*). The Toronto human knockout pooled library (TKOv3) was a gift from J. Moffat (Addgene, #125517) (*44*). These libraries were packaged into lentivirus and transduced into cells expressing Cas9 at an MOI (multiplicity of infection) of 0.3 with >150-fold guide coverage. Cells were pooled before any selection event.

For IRP2-Clover cell screens, cells were washed in PBS supplemented with 10 mM Hepes, passed through CellTrics 50-μm filters, and suspended in sort media (PBS with 10 mM Hepes and 2% FBS) on sort days, with the top 1% of fluorescent cells underwent FACS (BD Influx) into collection media (49% DMEM, 49% FBS, and 2% penicillin-streptomycin). Sorted cells were split for DNA extraction (Puregene Core Kit A, Qiagen, 158388) or expansion in recovery media (DMEM with 20% FBS and 1% penicillin-streptomycin) and then DMEM with 10% FBS, until a second sort of the enriched population, again selecting the top 1% of cells (Fig. 2A). An example gating strategy is shown in fig. S7. DNA was extracted from phenotypically nonselected library controls at each time point.

For intracellular antibody staining screens, cells were seeded 24 hours before sorting, with fixing and methanol permeabilization on sort days. Cells were stained with primary rabbit anti-IRP2 antibody at 1:200 and secondary AF488 anti-rabbit at 1:1000. Cells were then filtered before FACS (BD Influx) for the top 1% of fluorescent cells, which were collected and from which DNA was extracted (QIAmp DNA Mini Kit, Qiagen, 51304) (fig. S2A). A late time point sort was performed using cells that had not undergone FACS, again selecting the top 1% of fluorescent cells.

A two-stage PCR was completed to amplify inserts (table S6). Following PCR, DNA was purified (AMPure XP, Agencourt, A63880), quantified via Bioanalyzer (Agilent DNA 1000, 5067-1504), and sequencing performed by Illumina MiniSeq, HiSeq, or NovaSeq with a custom primer (table S6). DNA was extracted from phenotypically nonselected library controls at each time point.

A custom iron sublibrary containing 184 genes was developed with four top-ranking sgRNAs for each screen-identified gene generated using the Vienna Bioactivity CRISPR score (www.vbc-score.org) (*88*) (data S2). Chr10promiscuous, Luciferase, intergenic, and nontargeting control sgRNA sequences were taken from the TKO and Whitehead libraries (*42*, *43*). The sublibrary was purchased commercially (IDT) and amplified by PCR, with cleanup performed using the QIAquick Nucleotide Removal Kit (Qiagen 28306) per the manufacturer’s instructions. Guides were cloned into a pKLV backbone and transformed by electroporation into Stbl4 cells. After recovery, bacteria were serially diluted and plated overnight or continued in LB, both at 37°C. Colonies were counted to ensure representation and DNA extracted using a Maxi Kit before amplification in a two-stage PCR and sequencing via MiniSeq to confirm guide representation.

### CRISPR-Cas9 screen analysis

A Snakemake pipeline generated and curated by N.W. (https://github.com/niekwit/crispr-screens and https://doi.org/10.5281/zenodo.10286661) was used for bioinformatics analysis. Initially, the quality of the raw data was assessed using FastQC v0.12.1 and MultiQC v1.23. Reads were then quality trimmed to 20 base pairs (bp) using Cutadapt v4.0 (*89*). The trimmed reads were aligned to all sgRNA sequences using HISAT2. MAGeCK and BAGEL2 were used to determine the changes in normalized read counts between samples (*90*, *91*). This analysis compares DNA extracted after sorting to an unsorted DNA library. Outputs from MAGeCK and BAGEL2 are available in data S1.

### Chromatin immunoprecipitation sequencing

A Snakemake pipeline generated and curated by N.W. (https://github.com/niekwit/chip-seq and https://doi.org/10.5281/zenodo.138015-26) was used for bioinformatics analysis. After initial QC of the raw data [GSE110323 (*70*)] as above, reads were trimmed using Trim Galore v0.6.10. Bowtie2 v2.5.0 was used to align the reads to the human genome (hg38). From these BAM files, blacklisted regions were removed using BEDTools v2.31.1 (https://doi.org/10.1093/bioinformatics/btq033). BAM files were then sorted and duplicates removed using SAMtools v1.20 and Picard v3.2.0 (https://broadinstitute.github.io/picard/), respectively. To generate profile plots, deepTools v3.5.2 (https://doi.org/10.1093/nar/gkw257) subcommand plotProfile was used with BigWig files generated by the subcommand bamCoverage with BAM files as the input.

### RNA sequencing

Data analysis of publicly available RNA-seq data (GSE150609 and GSE110323) (*70*, *71*) was conducted using a Snakemake pipeline (https://doi.org/10.5281/zenodo.10139567). After assessing the quality of the raw data with FastQC and MultiQC, sequence reads were trimmed to remove adaptor sequences and low-quality nucleotides using TrimGalore. Transcript quantification was carried out with Salmon against the Gencode *Homo sapiens* reference transcriptome/genome (build 44) (*92*). Differential transcript analysis was performed using DESeq2, identifying genes with adjusted *P* values < 0.01 and log_2_(fold change) > 0.5 as differentially expressed for each comparison (*93*).

### Alternative splicing

Data analysis of publicly available RNA-seq data (GSE150609) was conducted (*71*). Trim Galore was used to remove adapter sequences. Read alignment was performed against the human genome (hg38) with STAR (*94*). To quantify alternative splicing events, rMATS was used (*95*). A custom R script using the package Tidyverse (https://doi.org/10.21105/joss.01686) was used for plotting.

### Gene ontology

Gene ontology analysis of screen hits was undertaken using g:Profiler (*45*).

### Inductively coupled plasma mass spectrometry

Inductively coupled plasma mass spectrometry (ICP-MS) was developed from a protocol by Stangherlin *et al.* (*96*) and performed in collaboration with J. Day (Department of Earth Sciences, University of Cambridge). For each biological replicate, ~5 × 10^5^ cells (corresponding to ~10 ng of iron) in technical triplicate were washed in PBS before lysis (30 min, RTP) with high-purity 65% nitric acid (Merck Millipore, 100441) supplemented with cerium [0.1 mg/liters (100 parts per billion), Romil, E3CE#] as a procedural internal standard. Before analysis, samples were diluted to a final concentration of 5% HNO_3_. Data were collected and analyzed using Syngistix version 1.1, normalized on the basis of cerium abundance and cell number (counted from duplicate cultures at time of harvest), and analyzed using GraphPad Prism.

### Ferroptosis assays

For manual cell counts, cells were seeded in 6-well plates, treated with erastin, and counted using Trypan blue after 48 hours using a hemocytometer. To assess lipid peroxidation, cells were seeded in 6-well plates and treated with erastin 24 hours before staining with 5 μM BODIPY 581/591 C11 (Thermo Fisher Scientific, D3861) for 35 min, before resuspension in 500 μl of Hanks’ balanced salt solution (Sigma-Aldrich, H9394) and analysis by flow cytometry using a 488-nm laser with a 530/30-nm band-pass filter (BD LSR II) and data processed using FlowJo (BD Life Sciences).

### NK competitive cytotoxicity assays

Primary human NK cells were isolated from leukocyte cones obtained from anonymous donors via NHS Blood and Transplant (REC 22/PR/1280 approved by London–Chelsea Research Ethics Committee) via negative selection using the NK Cell Isolation Kit (Miltenyi Biotec) and expanded for 14 days in NK MACS medium (Miltenyi Biotec) supplemented with 5% heat-inactivated human AB serum (Sigma-Aldrich), gentamicin sulfate (50 μg/ml; Thermo Fisher Scientific), 50 μM 2-mercaptoethanol (Gibco), and recombinant human interleukin-2 (IL-2) (500 U/ml, PeproTech) and IL-15 (10 U/ml, PeproTech). Assays were performed in peripheral blood mononuclear cell (PBMC) washing media [RPMI 1640 (Sigma-Aldrich)], supplemented with 10% heat-inactivated FBS (Sigma-Aldrich), gentamicin sulfate (50 μg/ml; Thermo Fisher Scientific), and 2 mM glutamine (Sigma-Aldrich).

Target cells were washed with DPBS and labeled with either carboxyfluorescein diacetate succinimidyl ester (CFSE) (0.5 nM, Invitrogen) or Tag-IT Violet Proliferation and Cell Tracking Dye (1.25 μM, BioLegend) for 20 min at 37°C. The reaction was quenched by the addition of 5 ml of PBMC washing media and incubated for 5 min at room temperature. After washing, the cells were counted and adjusted to the required density. The competitive cytotoxicity assay was set up in 96-well U-bottom plates with a total of 5 × 10^4^ target cells per well at a 1:1 ratio (control:*SETD2*sh) and a range of effector:target ratios as indicated in the figures. The plate was incubated at 37°C 5% CO_2_ for 4 hours. At the end of the assay, cells were washed with DPBS and stained with Fixable Viability Dye eFluor 780 (Thermo Fisher Scientific, 1:2000 dilution) for 5 min at 37°C. After washing with FACS buffer, cells were fixed with 2% PFA for 5 min at room temperature and washed once with FACS buffer before acquisition on a flow cytometer (MACSQuant VYB). Target cells were identified by CFSE or Violet and the percentage of dead cells with the Fixable Viability Dye eFluor 780. The specific cell death was calculated by subtracting the percentage of dead cells in the target only well.

### Statistical analysis

GraphPad Prism was used to calculate *P* values for individual experiments, including for paired *t* test and two-way analysis of variance (ANOVA) where indicated. CRISPR screens and ChIP-seq were analyzed as described in the relevant sections above. Kaplan-Meier curve data were analyzed using the log-rank test, generated by OncoLnc (*75*).
