# Supplementary material for: Mapping the genetic landscape of iron metabolism uncovers the SETD2 methyltransferase as a modulator of iron flux
Source: Sci Adv. 2025 Sep 17;11(38):eadw9095. doi: 10.1126/sciadv.adw9095 (PMC12442849; doi:10.1126/sciadv.adw9095)
Supplement: Supplementary file 1 — Figs. S1 to S10 Tables S1 to S6 Legends for data S1 to S3 [file sciadv.adw9095_sm.pdf]

Science Advances
AAAS

Supplementary Materials for

Mapping the genetic landscape of iron metabolism uncovers the SETD2 methyltransferase as a modulator of iron flux

Anthony W. Martinelli *et al.*

Corresponding author: James A. Nathan, jan33@cam.ac.uk

Sci. Adv. **11**, eadw9095 (2025)
DOI: 10.1126/sciadv.adw9095

The PDF file includes:

Figs. S1 to S10
Tables S1 to S6
Legends for data S1 to S3

Other Supplementary Material for this manuscript includes the following:

Data S1 to S3

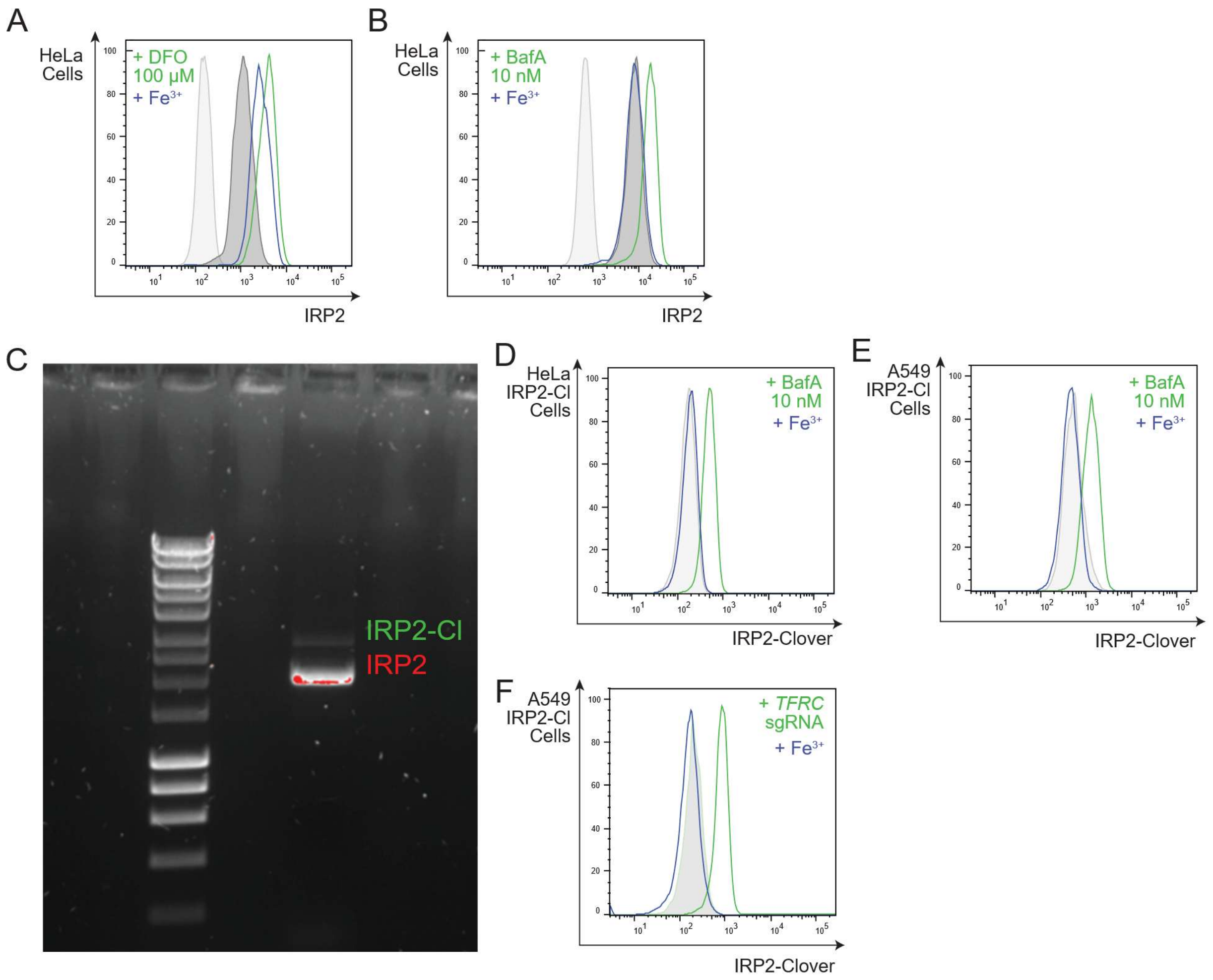

Fig. S1. IRP2 levels are a dynamic measure of iron flux.
(**A**, **B**) HeLa cells were treated with DFO (**A**, 100 µM, 20 hr) or BafA (**B**, 10 nM, 20 hr) ± iron supplementation (ferric citrate, 100 µM, 20 hr), fixed and permeabilised, and stained with primary anti-IRP2 antibody (1:200) and a secondary fluorescent antibody or a secondary antibody only control (light grey), prior to analysis by flow cytometry (n=3). (**C**) DNA from clonal A549 IRP2-Clover knock-in cells was amplified via PCR. Amplicon indicating insertion of Clover (Cl) is shown (IRP2-Cl). (**D**) HeLa IRP2-Cl cells were treated with BafA (10 nM, 20 hr) ± iron (FAC, 100µM, 20 hr) and analysed by flow cytometry (representative of at least 3 independent experiments). (**E**) A549 IRP2-Cl cells were treated with BafA (10 nM, 20 hr) ± iron (FAC, 100µM, 20 hr) and analysed by flow cytometry (representative of at least 3 independent experiments). (**F**) A549 IRP2-Cl cells were transduced with sgRNA targeting *TFRC* ± iron (FAC, 100µM, 20 hr) before analysis by flow cytometry (representative of at least 3 independent experiments). *DFO=desferrioxamine*, *BafA=bafilomycin A*, *IRP2-Cl=IRP2-Clover*.

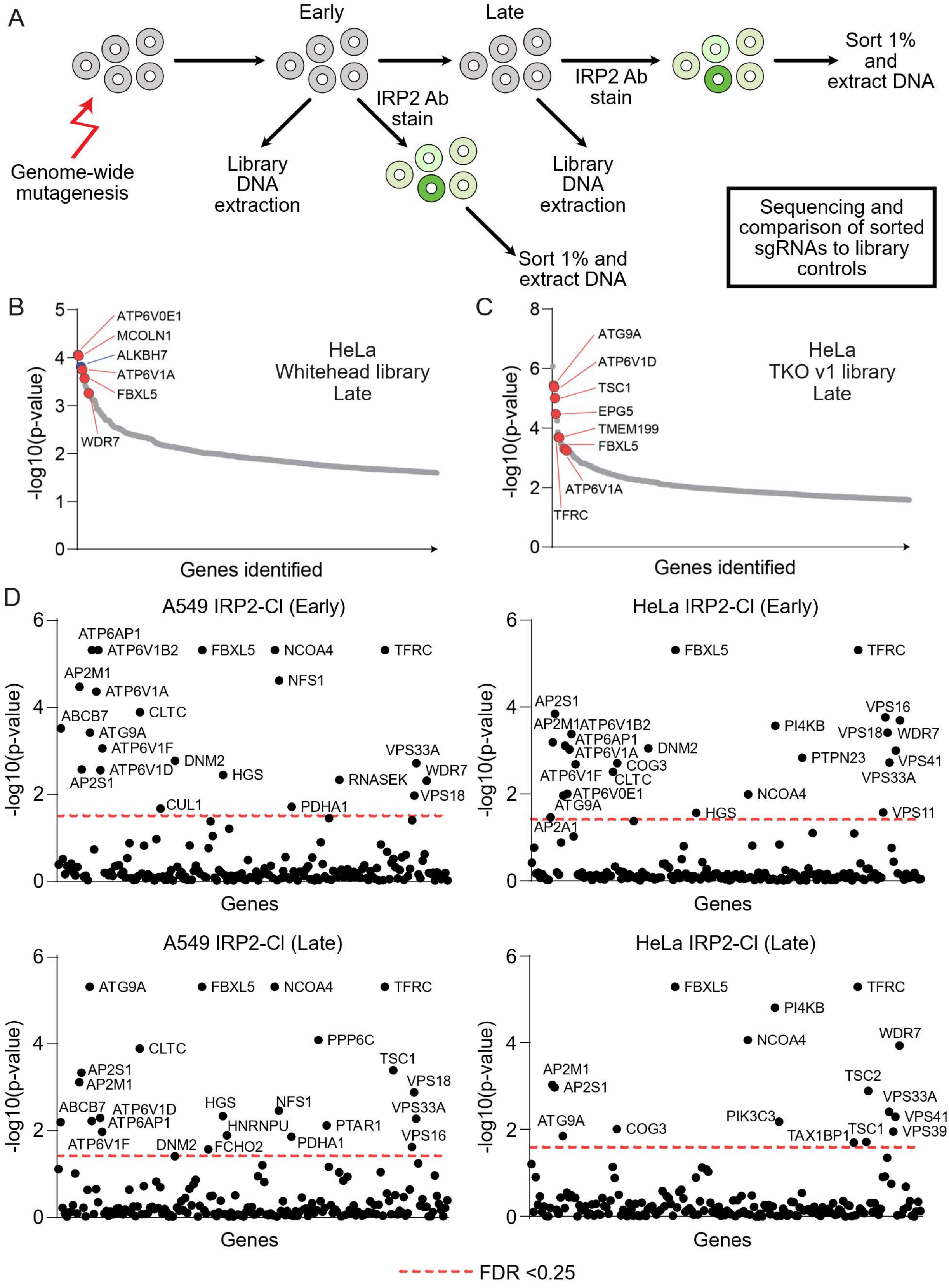

A
Early
Late
Genome-wide mutagenesis
Library DNA extraction
IRP2 Ab stain
Sort 1% and extract DNA
Sequencing and comparison of sorted sgRNAs to library controls
B
HeLa Whitehead library Late
ATP6V0E1
MCOLN1
ALKBH7
ATP6V1A
FBXL5
WDR7
-log10(p-value)
Genes identified
C
HeLa TKO v1 library Late
ATG9A
ATP6V1D
TSC1
EPG5
TMEM199
FBXL5
ATP6V1A
TFRC
D
A549 IRP2-CI (Early)
HeLa IRP2-CI (Early)
A549 IRP2-CI (Late)
HeLa IRP2-CI (Late)
Genes
FDR <0.25

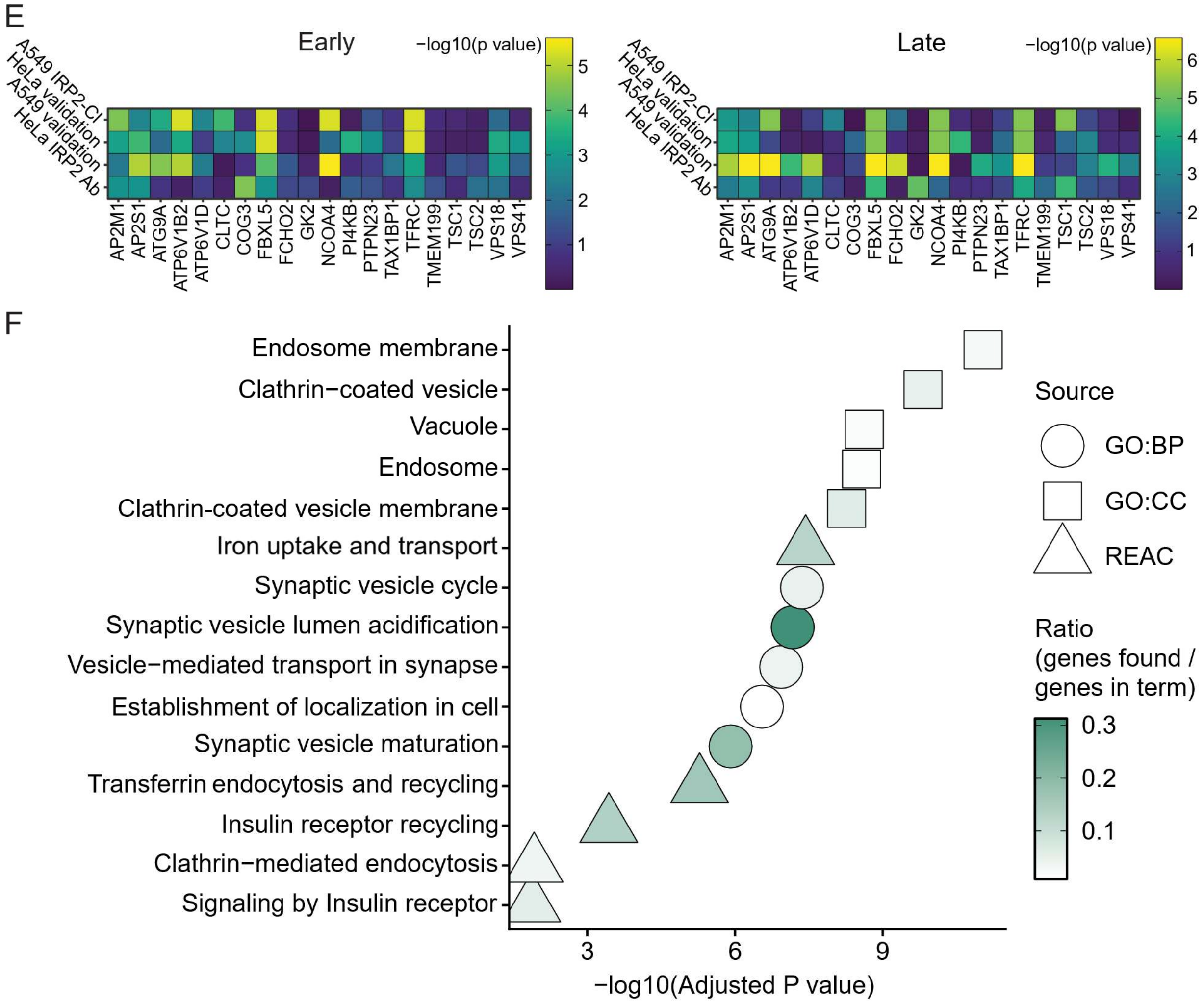

Fig. S2. CRISPR screens identify key genes and pathways in iron metabolism.
(**A**) Schematic depicting workflow for an intracellular antibody staining forward genetic screen. (**B**, **C**) Whitehead (**B**) or TKOv1 (**C**) genome-wide CRISPR libraries were transduced into HeLa cells. DNA extracted from top 1% IRP2^{HIGH} cells by FACS was extracted and compared to library controls by MAGeCK. Bubble plots illustrate top candidate genes by -log10(p-value) at a late time point sort (day 14-15). (**D**) A549 IRP2-Cl and HeLa IRP2-Cl cells were transduced with a sub-pooled validation library composed targeting 188 candidate genes from initial mutagenesis screens. Analysis of DNA from sorted IRP2HIGH cells compared to non-selected controls was undertaken using MAGeCK. Results are presented by -log10(p-value) (y-axis) and gene name (x-axis), with hits below a false discovery rate of 0.25 also illustrated and both early and late time points. (**E**) Heat maps illustrating relative -log(p-value) selected hits across primary screens in HeLa (antibody screen, TKOv1) and A549 IRP2-Clover (TKOv3) and secondary validation sub-pooled library screens in HeLa and A549 IRP2-Clover cells. (**F**) Gene ontology analysis of top hit candidate genes from the A549 IRP2-Clover TKOv3 screen, generated using g:Profiler. *FDR=false discovery rate*, *GO:BP=Gene Ontology: Biological Process*, *GO:CC=Gene Ontology: Cellular Compartment*, *REAC=reactome.*

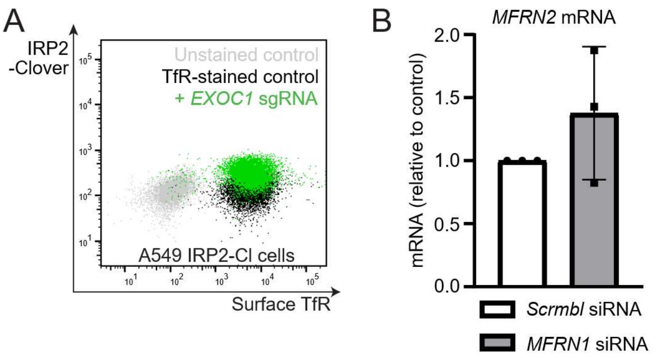

Fig. S3. The exocyst complex and mitochondrial iron import affect cytosolic iron availability.
(**A**) A549 IRP2-Clover cells were transduced with sgRNA targeting *EXOC1* and stained with antibody targeting surface TfR before analysis by flow cytometry (n=2). (**B**) A549 IRP2-Clover cells were transfected with siRNA targeting *MFRN1* and *MFRN2* mRNA expression measured by RT-qPCR (n=3). *IRP2-Cl=IRP2-Clover*.

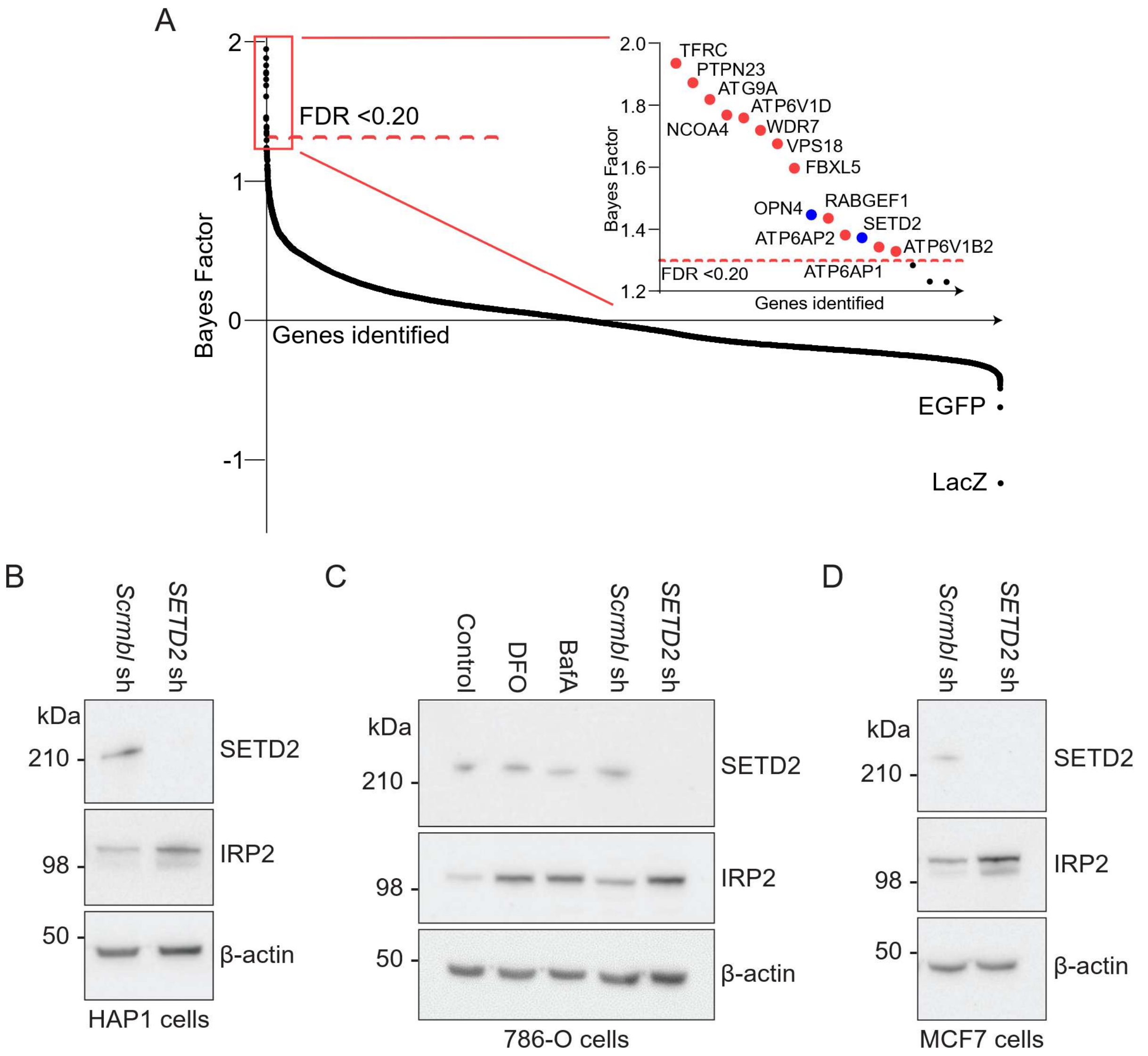

Fig. S4. SETD2 loss results in IRP2 accumulation across cell lines.
(**A**) BAGEL2 analysis of A549 IRP2-Clover TKOv3 CRISPR screen. DNA extracted from IRP2-Clover^{HIGH} cells at the day 16 second sort was compared to library controls. Inset magnifies top 20 genes (FDR <0.20). EGFP and LacZ are controls within the TKOv3 sgRNA library. (**B**, **C**, **D**) HAP1 (n=3) (**B**), 786-O (n=3) (**C**), and MCF7 (n=2) (**D**) cells were transduced with shRNA targeting *SETD2* or a scrambled shRNA control, and protein levels of SETD2, IRP2, and β-actin were measured by immunoblot. *BAGEL2=Bayesian Analysis of Gene EssentiaLity 2*, *FDR=false discovery rate*, *DFO=desferrioxamine*, *BafA=bafilomycin A*.

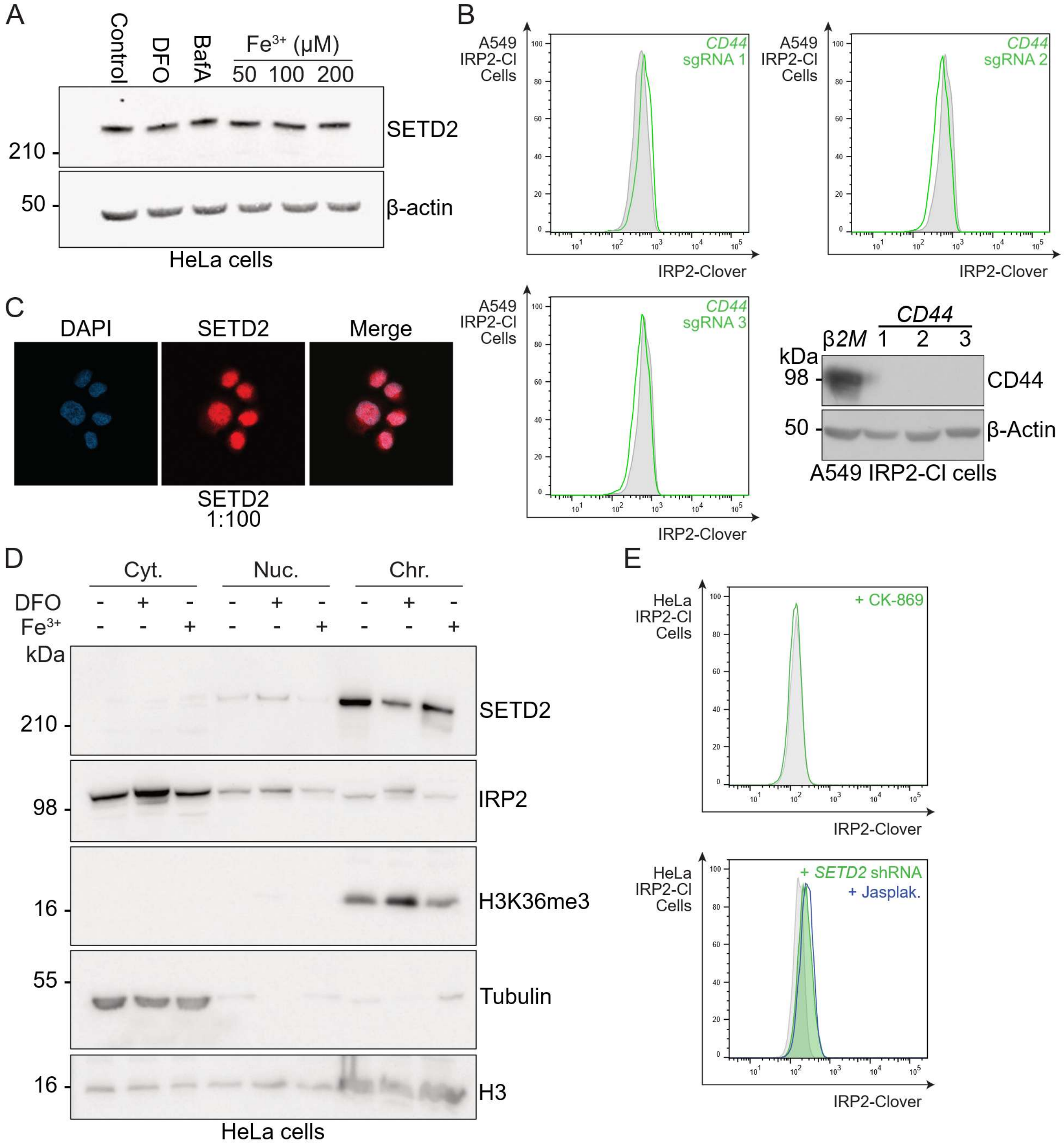

Fig. S5. IRP2 accumulation in SETD2 loss is mediated via the nucleus.
(**A**) HeLa cells were treated with DFO (100 μM, 20 hr), BafA (10 nM, 20 hr) or ferric iron excess (FAC, 50-200 μM, 20 hr) and analysed by immunoblot for SETD2 and β-actin (n=3). (**B**) A549 IRP2-Clover cells were transduced with three separate sgRNAs targeting *CD44* and analysed by flow cytometry, each using the same non-transduced control. Knockout of *CD44* was confirmed by immunoblot for CD44 and β-actin (n=1 for each guide). (**C**) Micrograph of HeLa cells stained with primary anti-SETD2 antibody (1:100) and DAPI (n=2). (**D**) HeLa cells were treated with iron chelation (DFO, 100 μM, 20 hr) or ferric iron excess (FAC, 200 μM, 20 hr) before subcellular fractionation. Cytosolic, nucleosolic, and chromatin fractions were analysed by immunoblot for SETD2, IRP2, H3K36me3, Tubulin, and H3 (n=3). (**E**) HeLa IRP2-

Clover cells were treated with CK-869 (left panel; 200 nM, 24 hr) or transduced with shRNA targeting *SETD2* followed by treatment with Jasplakinolide (right panel; 100 nM, 24 hr) before analysis by flow cytometry (n=3). *DFO=desferrioxamine*, *BafA=Baifliomycin A*, *IRP2-Cl=IRP2-Clover*, *Jasplak.=jasplakinolide*.

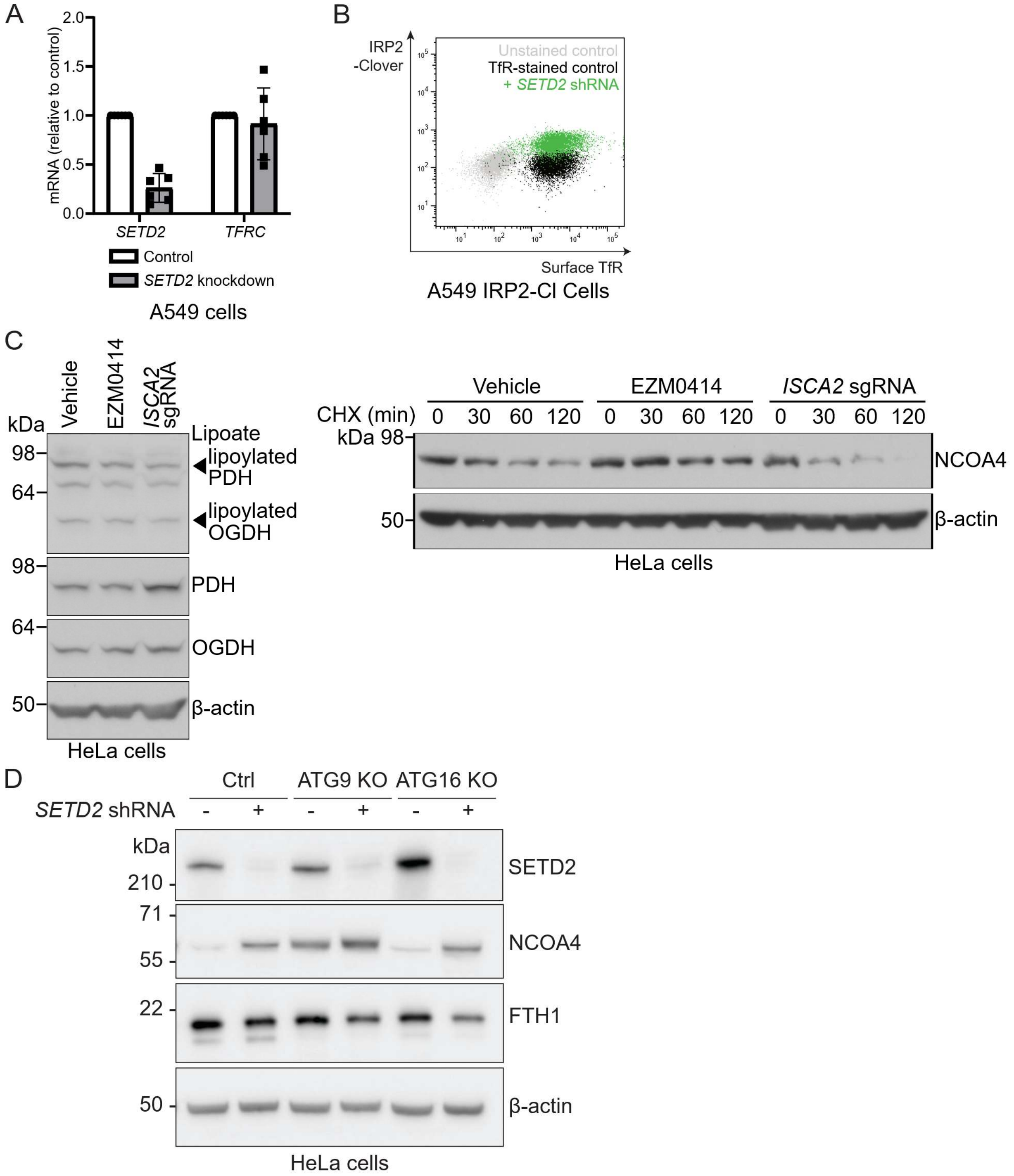

Fig. S6. SETD2 loss impacts ferritinophagy.
(**A**) A549 IRP2-Clover cells were transduced with shRNA targeting *SETD2*. Transcript levels of *SETD2* and *TFRC* were measured by RT-qPCR (n=6). (**B**) Cell surface staining for TfR was performed and surface TfR and IRP2-Clover levels were measured by flow cytometry (n=3). (**C**) HeLa cells were transduced with sgRNA targeting *β2M* (control) or *ISCA2* for 11 days, then treated with 0.4% (v/v) DMSO or 200 nM EZM0414 for 48 hr. Cells were then treated with 10

ng/µl cycloheximide (CHX) for the indicated times prior to analysis by immunoblot for NCOA4, Lipoate, pyruvate dehydrogenase (PDH), 2-oxoglutarate dehydrogenase (OGDH), and β-actin (n=4). PDH and OGDH are two lipoylated enzymatic complexes within the mitochondrion. (**D**) HeLa null *ATG9A*, *ATG16*, or paired controls were transduced with shRNA targeting *SETD2* and immunoblot performed for SETD2, NCOA4, FTH1 and β-actin (representative of at least three biological replicates). *IRP2-Cl=IRP2-Clover*.

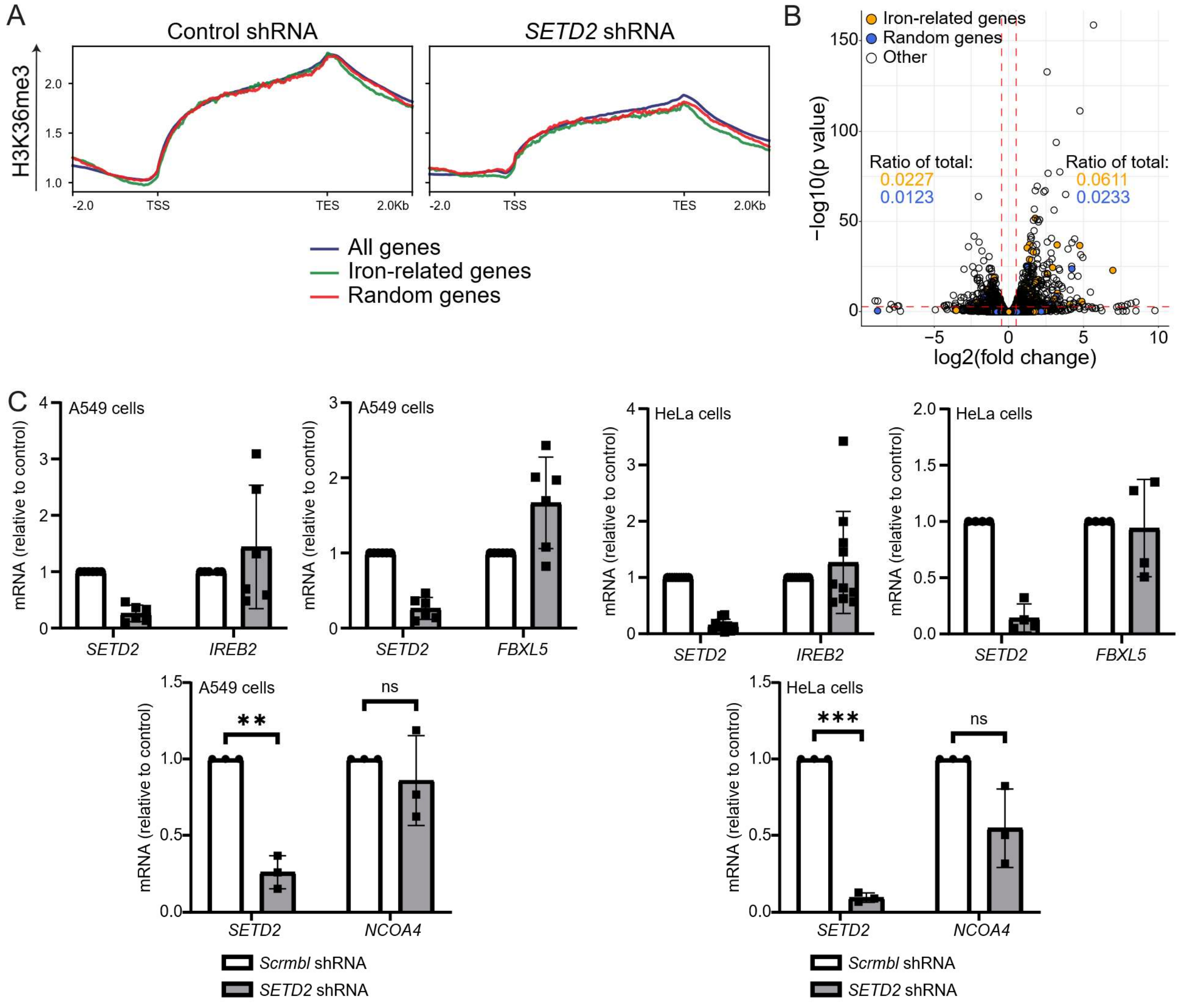

Fig. S7. Genes known to regulate iron metabolism are not differentially transcribed in SETD2 loss.

(**A**) ChIP-seq analysis of publicly available data from HepG2 cells treated with *SETD2* or control knockdown. H3K36me3 is shown for all genes (blue) and a custom datasets of iron-related gense (green, 731 genes). A group of randomly selected genes equal to the number of iron-related genes was included as a control (red). Data extracted from GSE110323. (**B**) RNA-seq analysis of publicly available data from HepG2 cells treated with *SETD2* or control knockdown. Volcano plots showing up or down-regulated transcripts with highlighted custom datasets of iron-related genes (orange, 731 genes). A group of randomly selected genes equal to the number of iron-related genes was included as a control (blue). Data extracted from GSE110323. (**C**) A549 or HeLa cells with transduced with shRNA targeting *SETD2* or a scrambled control, and RT-qPCR was undertaken to assess transcript levels of *SETD2*, *IREB2* (n=6 and n=10), *FBXL5* (n=6 and n=4) and *NCOA4* (n=3 and n=3). *TSS=transcription start site*, *TES=transcription end site.*

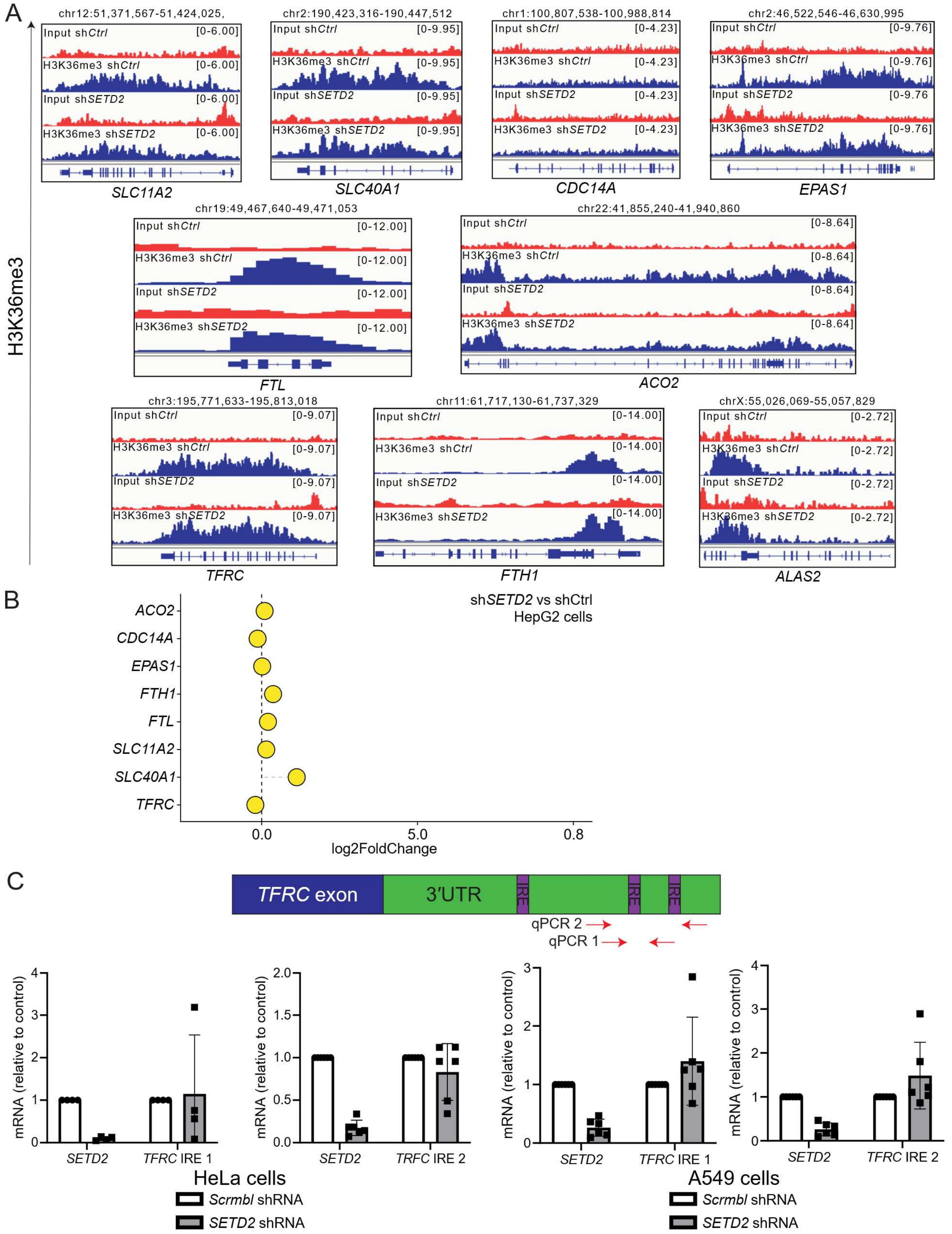

Fig. S8. Known iron responsive elements are unaffected by SETD2 loss.

(**A**, **B**) ChIP-seq (**A**) and RNA-seq (**B**) analysis of publicly available data from HepG2 cells treated with *SETD2* or control knockdown. H3K36me3 or transcript expression relative to controls is shown for genes known to contain IREs. Data extracted from GSE110323. (**C**) Schematic showing design of primers targeting IREs within *TFRC*. HeLa and A549 cells were transduced with shRNA targeting *SETD2* or a scramble control and RT-qPCR undertaken to measure levels of *SETD2* and both IRE-adjacent regions of *TFRC* transcript (n≥4).

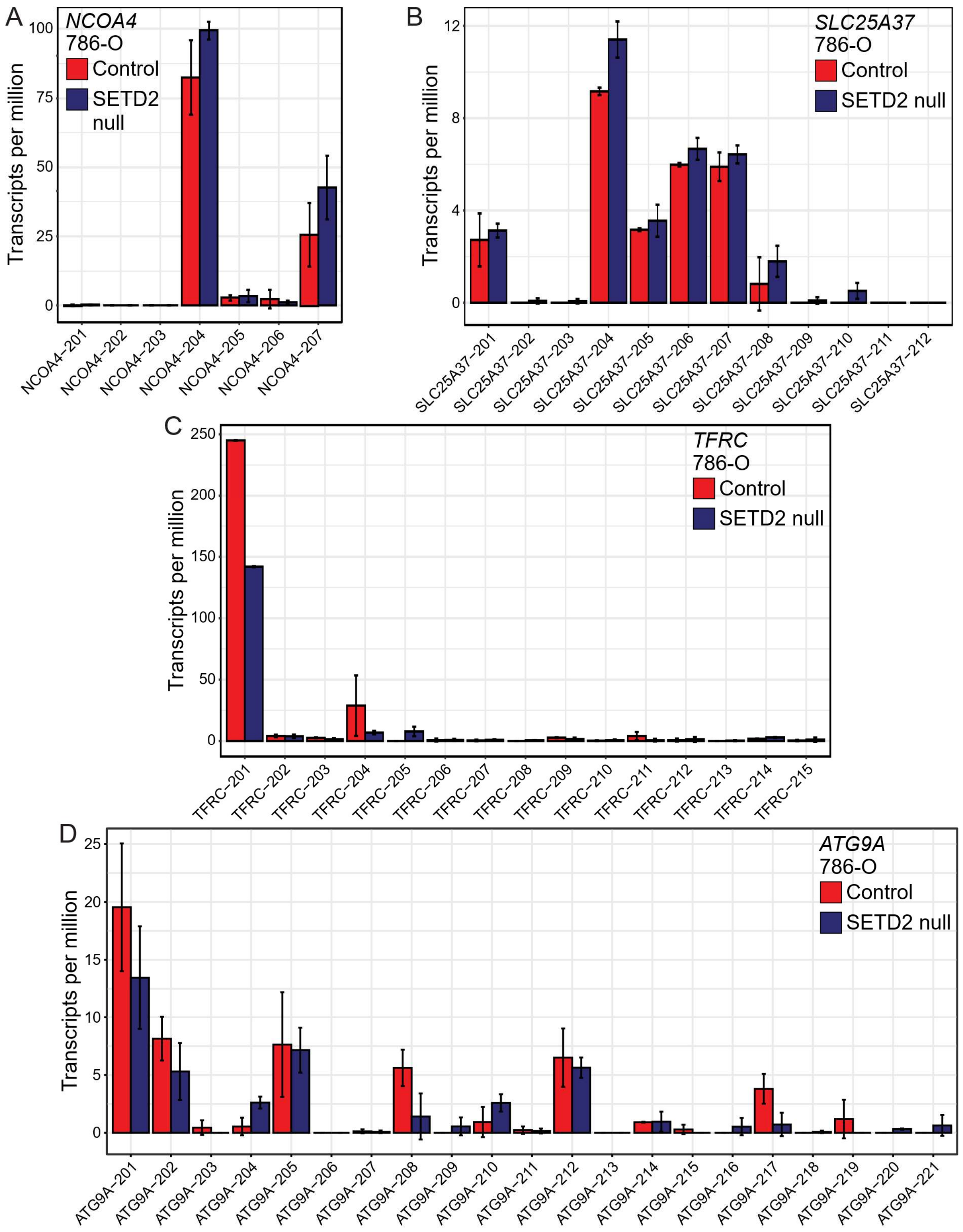

A
NCOA4
786-O
Control
SETD2 null
Transcripts per million
0
25
50
75
100
NCOA4-201
NCOA4-202
NCOA4-203
NCOA4-204
NCOA4-205
NCOA4-206
NCOA4-207
B
SLC25A37
786-O
Control
SETD2 null
Transcripts per million
0
4
8
12
SLC25A37-201
SLC25A37-202
SLC25A37-203
SLC25A37-204
SLC25A37-205
SLC25A37-206
SLC25A37-207
SLC25A37-208
SLC25A37-209
SLC25A37-210
SLC25A37-211
SLC25A37-212
C
TFRC
786-O
Control
SETD2 null
Transcripts per million
0
50
100
150
200
250
TFRC-201
TFRC-202
TFRC-203
TFRC-204
TFRC-205
TFRC-206
TFRC-207
TFRC-208
TFRC-209
TFRC-210
TFRC-211
TFRC-212
TFRC-213
TFRC-214
TFRC-215
D
ATG9A
786-O
Control
SETD2 null
Transcripts per million
0
5
10
15
20
25
ATG9A-201
ATG9A-202
ATG9A-203
ATG9A-204
ATG9A-205
ATG9A-206
ATG9A-207
ATG9A-208
ATG9A-209
ATG9A-210
ATG9A-211
ATG9A-212
ATG9A-213
ATG9A-214
ATG9A-215
ATG9A-216
ATG9A-217
ATG9A-218
ATG9A-219
ATG9A-220
ATG9A-221

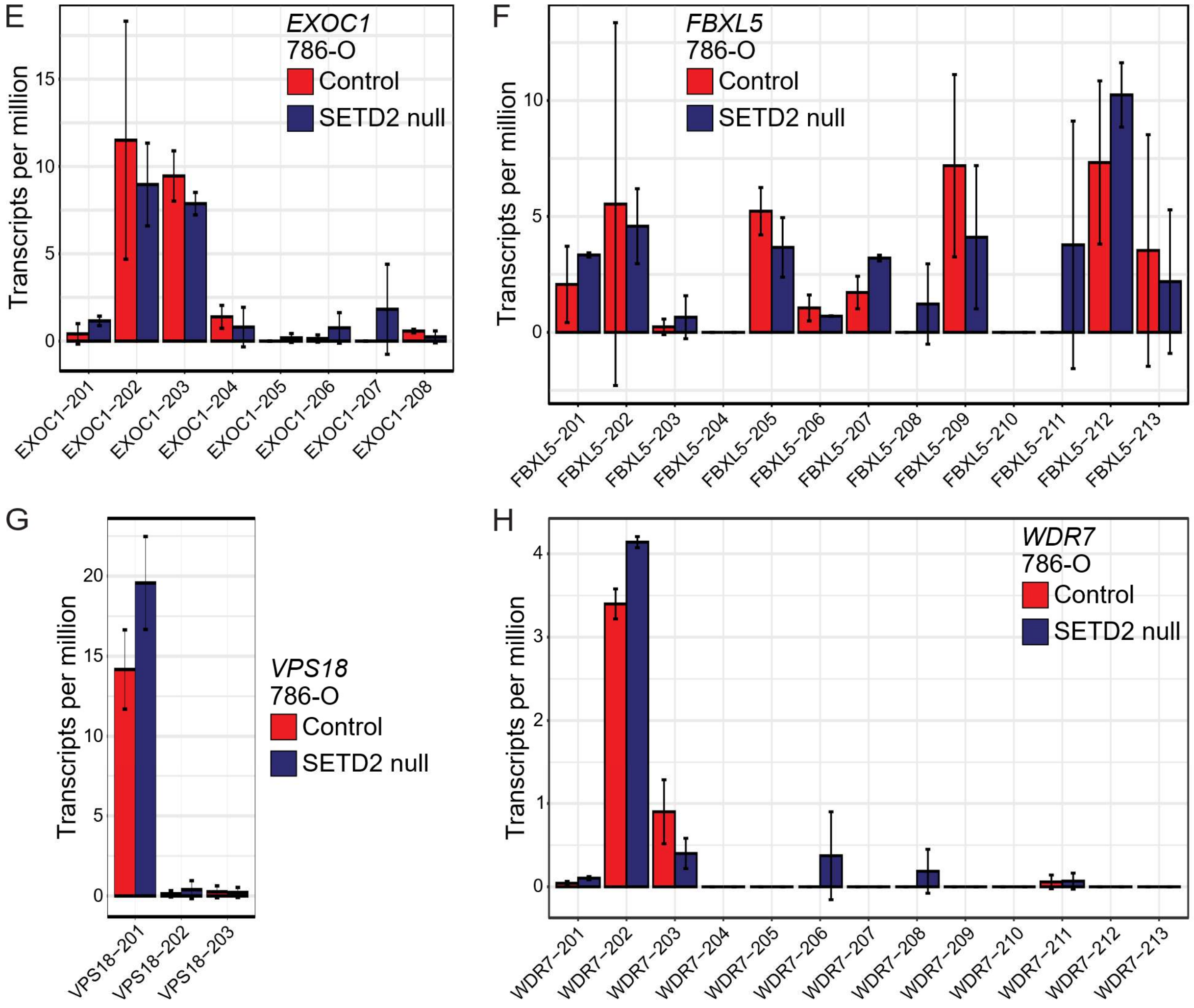

Fig. S9. Isoforms of NCOA4 are differentially expressed in SETD2 loss.
(**A-H**) RNA-seq analysis of publicly available data from wild type and SETD2 stable knockout (SETD2 null) 786-O cells. Transcript isoform abundance was analysed using Salmon for (**A**) *NCOA4* (p=0.0459 for column factor, two-way ANOVA), (**B**) *SLC25A37* (Mitoferrin-1; p=0.0011 for column factor, two-way ANOVA), (**C**) *TFRC* (p<0.0001 for column factor, two-way ANOVA), (**D**) *ATG9A* (p=0.0716 for column factor, two-way ANOVA), (**E**) *EXOC1* (p=0.7702 for column factor, two-way ANOVA), (**F**) *FBXL5* (p=0.6894 for column factor, two-way ANOVA), (**G**) *VPS18* (p=0.0870 for column factor, two-way ANOVA), and (**H**) *WDR7* (p=0.1183 for column factor, two-way ANOVA). Data extracted from GSE150609.

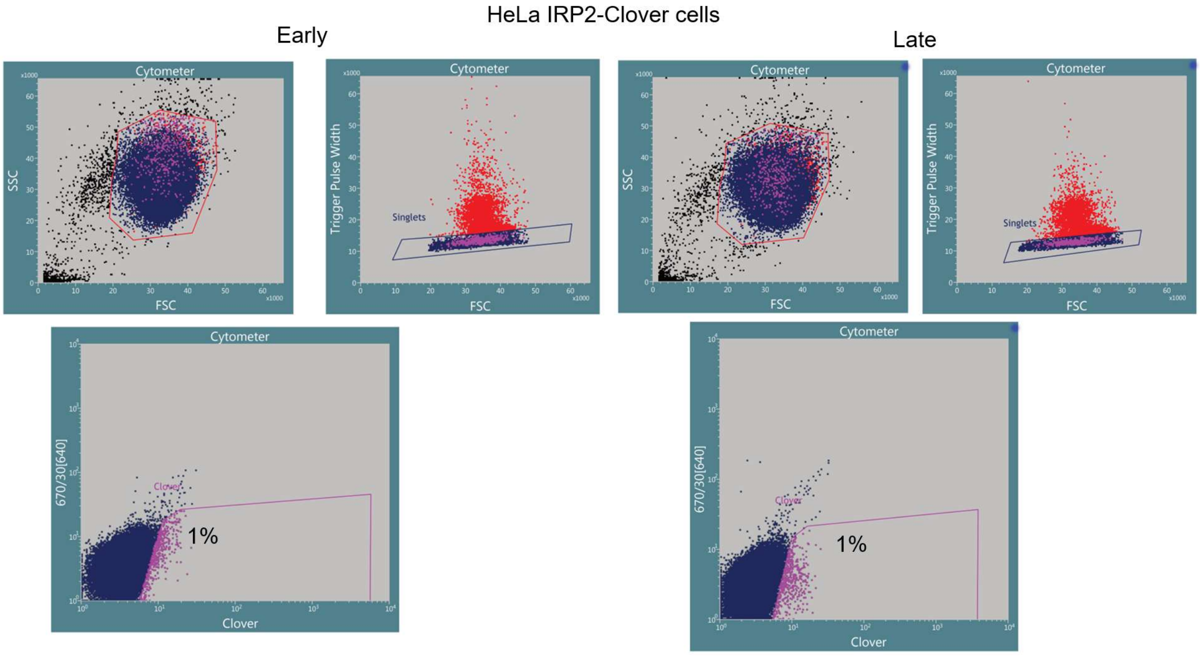

Fig. S10. Example gating strategy for a CRISPR/Cas9 forward genetic screen.
Data from HeLa IRP2-Clover screen with Whitehead library at early and late time points. *SSC=side scatter*, *FSC=forward scatter*.

Antibody	Source	Identifier	Dilution
β-actin	Sigma	A228	IB: 1:30,000
CD44	Abcam	ab189524	IB: 1:10,000
CD71/TfR1	BD Pharmigen	555534	FC: 1:500
FTH1	Cell Signaling	#3998	IB: 1:1,000
Histone H3 (D1H2)	Cell Signaling	#4499	IB: 1:2,000
H3K36me3	Cell Signaling	#9763	IB: 1:1,000
IRP2 (D6E6W)	Cell Signaling	#37135	IB: 1:1,000
Lipoate	Sigma-Aldrich	437695	IB: 1:1,000
NCOA4 (ARA70)	Bethyl	A302-272A	IB: 1:1,000
OGDHc-E2	Cell Signaling	11954	IB: 1:,2000
PDHc-E2	Cell Signaling	12362	IB: 1:,2000
SETD2	ABclonal	A3194	IB: 1:1,000<br>IF: 1:100
HRP anti-mouse	Jackson	115-035-146	IB: 1:20,000
HRP anti-rabbit	Jackson	115-035-045	IB: 1:20,000
Alexa Fluor 488 (anti-rabbit)	Invitrogen	A11034	FC: 1:1,000<br>IF: 1:400
Alexa Fluor 568 (anti-rabbit)	Invitrogen	A11036	IF: 1:400
Alexa Fluor 647 (anti-rabbit)	Invitrogen	A21245	IF: 1:400
Alexa Fluor 647 (anti-mouse)	Invitrogen	A11036	FC: 1:1000

Table S1. List of antibodies. *IB=immunoblot*, *IF=immunofluorescence*, *FC=flow cytometry*.

Function	Sequence
Knock-in 5′ forward	TTGGGCTCCCCGGGCGCGACTAGTGAATTCCTCC TGGAATTACATTGAATATACAGGTAT
Knock-in 5′ reverse	ACCAGATCCGCCACCAGATCCGCCCGATCGTGAG AATTTTCGTGCTACAAAGTTTAATAA
Knock-in 3′ forward	AGCATACATTATACGAAGTTATTTAATTAATATCTAC TTACAATAGATACGTTTCATAAC
Knock-in 3′ reverse	TTCTTATAATCAGCATCATGATGTGGTACCGTTCAG AGTTTAATGATTGAATAACATTCT
IREB2 sgRNA	ATTCCTGGGTCCAGCACAAA
Sequencing forward	CTCCTGGAATTACATTGAATATACAGGTAT
Sequencing reverse	GTTCAGAGTTTAATGATTGAATAACATTCT

Table S2. List of sequences for IRP2-Clover knock-in.

Target	Sequence (5’ → 3’)
EXOC1 (sh)	GATGAATACCAAGAGTTAAAT
HSCB (si)	CATAGAAATAATGGAAATCAA
MFRN1 (si)	GGUAAUGAAUCCAGCAGAA
Scrmbl (sh) (1)	GCATAATTAATATCCGCGTGT
Scrmbl (sh) (2)	ATGGATATATAACGACCTAGT
Scrmbl (sh) (3)	ATACGATGAATAGACGACAGC
Scrmbl (si)	CAGUCGCGUUUGCGACUGG
SETD2 (1) (sh)	AGTAGTGCTTCCCGTTATAAA
SETD2 (2) (sh)	ACGAATTAAAGACCGCAATAA

Table S3. List of knockdown sequences.

Primer name	Sequence (5’ → 3’)
ACTB forward	CTGGGAGTGGGTGGAGGC
ACTB reverse	TCAACTGGTCTCAAGTCAGTG
EXOC1 forward	CCTGCTGAGCATTGTGAATGT
EXOC1 reverse	CACAGGGCGTTCAGTTGTCA
FBXL5 forward	AGAACACTCCACAGGTATAACCC
FBXL5 reverse	CTGCATCGACATAACTCTTGAGG
HSCB forward	AGAGAAGCATTCGACCCTGGT
HSCB reverse	AGGAATTGCCTGTCCATTTCAT
IREB2 forward	TCGATGTATCTAAACTTGGCACC
IREB2 reverse	GCCATCACAATTTCGTACAGCAG
MFRN1 forward	GATGGGGACAGCCGAGATG
MFRN1 reverse	ACCGGGTACATGACCGAGT
MFRN2 forward	GTACCCCATCGACTGCGTC
MFRN2 reverse	CTCCAACACATTGCGATAGCG
NCOA4 forward	CAGCAGCTCTACTCGTTATTG G
NCOA4 reverse	TCTCCAGGCACACAGAGACT
SETD2 forward	TGCTTCTAGTCGATTTTTGCCC
SETD2 reverse	AGGGTTTGGAGTATCACTTTGC
TFRC forward	ACCGGCACCATCAAGCT
TFRC reverse	TGATCACGCCAGACTTTGC
TFRC IRE1 forward	TCCAGTACCTTTGTCACAATCCT
TFRC IRE1 reverse	TCCGATACAGACACTGTGGT
TFRC IRE2 forward	TCCAAGGTGTAACTCTAATTCCCA
TFRC IRE2 reverse	TGTTCCCGATAATTACTTACACCC

Taable S4. List of qPCR primers. Sequences generated via PrimerBank (*85*) or previously published (*86*).

Target	Sequence (5’ → 3’)
B2M	GGCCGAGATGTCTCGCTCCG
CD44 (1)	GAAAGCCTTGCAGAGGTCAG
CD44 (2)	ATGGAGAAAGCTCTGAGCAT
CD44 (3)	TCGCTACAGCATCTCTCGGA
EXOC1 (1)	GCATACACCAAACTTATCAG
EXOC1 (2)	GGCTAACATCCAGTCAATCA
ISCA2	CGTTAGGGACGACCCCCAGG
NCOA4	CAATCTCCACACCTTTGGGC
SETD2 (1)	GCGGAGCTGATACTTACTCA
SETD2 (2)	GGACTGTGAACGGACAACTG
TFRC	GCTCTGGAGATTGTCTGGAC

Table S5. List of sgRNA sequences.

Function	Sequence
Whitehead/TKOv1 outer PCR forward	AGGGCCTATTTCCCATGATTCCTT
Whitehead/TKOv1 outer PCR reverse	TCAAAAAAGCACCGACTCGG
TKOv3 outer PCR forward	GAGGGCCTATTTCCCATGATTC
TKOv3 outer PCR reverse	CAAACCCAGGGCTGCCTTGGAA
Inner PCR forward	AATGATACGGCGACCACCGAGATCTACACTCTCTTG TGGAAAGGACGAGGTACCG
Inner PCR reverse	CAAGCAGAAGACGGCATACGAGATNNNNNNGTGA CTGGAGTTCAGACGTGTGCTCTTCCGATCTATTTTA ACTTGCTATTTCTAGCTCTAAAAC
Sequencing primer	ACACTCTCTTGTGGAAAGGACGAAACACCG

Table S6. List of sequences for CRISPR/Cas9 screens.

Data S1. (separate file)

Tab 1: MAGeCK output for A549 IRP2-Clover TKOv3 screen (early time point). **Tab 2** MAGeCK output for A549 IRP2-Clover TKOv3 screen (late time point). **Tab 3:** MAGeCK output for HeLa IRP2-Clover Whitehead screen (early time point). **Tab 4:** MAGeCK output for HeLa IRP2-Clover Whitehead screen (late time point). **Tab 5:** MAGeCK output for HeLa IRP2 antibody Whitehead screen (late time point). **Tab 6:** MAGeCK output for HeLa IRP2 antibody TKOv1 screen (late time point). **Tab 7:** MAGeCK output for A549 IRP2-Clover iron sublibrary screen (early time point). **Tab 8:** MAGeCK output for A549 IRP2-Clover iron sublibrary screen (late time point). **Tab 9:** MAGeCK output for HeLa IRP2-Clover iron sublibrary screen (early time point). **Tab 10:** MAGeCK output for HeLa IRP2-Clover iron sublibrary screen (late time point). **Tab 11:** BAGEL2 output for A549 IRP2-Clover TKOv3 screen (late time point). *MAGeCK=Model-based Analysis of Genome-wide CRISPR-Cas9 Knockout*, *BAGEL2=Bayesian Analysis of Gene EssentiaLity 2*.

Data S2. (separate file)

Tab 1: Genes included in sub-pooled sgRNA library. Control sgRNAs from other CRISPR libraries: chr10Promiscuous (TKO), Control (Whitehead), Intergenic (Whitehead), Luciferase (TKO). **Tab 2:** List of iron-related genes, defined by GO designation.

Data S3. (separate file)

Tab 1: Top genes from A549 IRP2-Clover and HeLa IRP2-Clover screens (**data S2 Tabs 1-4**) including those with a -log(p-value) ≥3, annotated where mapped in **Fig. 2F**.
